# AI-integrated metabolomics maps functional divergence of microbial consortia in field-grown maize

**DOI:** 10.1007/s00299-025-03600-z

**Published:** 2025-09-11

**Authors:** Musiwalo Samuel Mulaudzi, Lerato Pertunia Nephali, Fidele Tugizimana

**Affiliations:** 1 Research Centre for Plant Metabolomics, Department of Biochemistry, University of Johannesburg, Auckland Park, Johannesburg, South Africa; 2 International Research and Development Division, Omnia Group, Ltd., Bryanston, Johannesburg, 2021 South Africa

**Keywords:** Artificial intelligence, Microbial biostimulants, Sustainable agriculture, Computational metabolomics, Biochemical and molecular mechanisms

## Abstract

**Key message:**

The study provides actionable insights into modes of action of the three microbial biostimulants on maize plants under field conditions. The postulated framework indicates a divergence model involving (i) photoprotection, structural reinforcement, and defense priming, (ii) systemic metabolic reprograming for growth and defense, and (iii) hormonal signalling modulation for stress response. These insights offer a data-driven blueprint for the next generation of sustainable, effective, and field-ready bioformulations.

**Abstract:**

Microbial consortia are currently recognized as a promising strategy for sustainable agriculture due to their ability to enhance plant growth, improve soil health, and mitigate environmental stresses. However, the biochemical and molecular mechanisms governing these beneficial effects on crops under field conditions remain poorly understood, and differential effects due to different microbial formulations are enigmatic. This study, therefore, aims to unravel the metabolic alterations, in maize plants, induced by three microbial biostimulants, under field conditions at different growth stages. Leaves from biostimulant-treated and untreated control maize plants were harvested at different time points. Metabolites were extracted using methanol. The extracts were analyzed on LC–MS/MS system. Computational metabolomics workflows and AI-driven strategies such as molecular networking and machine learning methods (PCA and OPLS-DA) were applied to mine and interpret spectral data. Machine learning models revealed the common and unique significant metabolites among the consortia at the vegetative stage. One of the key findings was that hydroxycinnamic acid (HCA) derivatives are the discriminatory metabolites differentiating the effects of the three microbial consortia on maize plants. Moreover, the results showed that consortia application significantly impacted primary and secondary maize metabolism, reprogramming biological pathways such as phenylalanine, tyrosine, and tryptophan biosynthesis, tyrosine metabolism, the citrate cycle (TCA cycle), flavone and flavonol biosynthesis, and flavonoid biosynthesis. These pathways are associated with plant defense, priming and development. Thus, this study sheds light on the complex molecular interactions between maize and microbial biostimulants under real-world conditions. It reveals that distinct microbial formulations differentially influence plant metabolism by reprogramming defense- and growth-related pathways. These actionable insights establish a foundational framework for functionally characterizing microbial consortia and pave the way for the rational design of next generation biostimulants tailored to specific crop needs and growth stages.

**Supplementary Information:**

The online version contains supplementary material available at 10.1007/s00299-025-03600-z.

## Introduction

The agricultural sector faces rising productivity challenges to feed the growing global population and increase resource use efficiency while reducing the environmental impact on ecosystems and human health (Rouphael and Colla 2020). The use of chemical fertilizers has long been the preferred strategy to boost crop yields. However, research and empirical evidence have shown that this traditional method is non-sustainable due to its negative impact on the environment (Etesami et al. 2023). As such, one of the innovative strategies and solutions to address these challenges in the agricultural sector, and for sustainable global food security, is the effective use and development of plant biostimulants (Rouphael and Colla 2018). These formulations include both microbial and non-microbial biostimulants. Microbial biostimulants stimulate physiological and biochemical processes that benefit nutrient uptake, nutrient efficiency, increase tolerance to abiotic stress, crop quality, and yield of plants (Castiglione et al. 2021; Shahrajabian et al. 2023). Microbial biostimulants can be categorized as either single microorganism formulations or microbial consortia and are normally applied to plants via seed treatment, foliar spraying, or rhizosphere application (Shahrajabian et al. 2023). Several plant-associated Bacillus species have been commercialized as biostimulants (Tsotetsi et al. 2022).

The use of microbial consortia as biostimulants in agriculture holds promise for enhancing crop productivity and resilience against environmental stresses. However, the mechanistic understanding of how these biostimulants exert their beneficial effects at the cellular and molecular levels remains limited, posing challenges to their innovative formulations and effective application in agricultural practices. Metabolomics has emerged as a pivotal approach in elucidating the molecular events and mechanisms underlying the effects of biostimulant formulations on plants (Nephali et al. 2020). Metabolomics is a multidisciplinary ‘omics’ science involving the comprehensive qualitative and quantitative characterization of metabolites in a biological matrix (Hong et al. 2016; Tebani et al. 2018). Recent studies have shown metabolomics capabilities in revealing the biochemical events triggered by biostimulants (Nephali et al., (2021; Bonini et al. 2020).

However, it is worth noting that most studies examining biostimulants’ effects on crop plants are predominantly conducted under greenhouse and controlled conditions. Despite the valuable insights gained from greenhouse studies, there is a pressing need for research conducted under field conditions. In real-world settings, plants are simultaneously exposed to a combination of various abiotic and biotic stressors, including soil variability, pest pressures, and fluctuating weather patterns. Such factors can reduce the efficacy of the microbial biostimulant under field conditions (Li et al. 2022; Mendes et al. 2023; Wadduwage et al. 2024). As such, field trials are necessary steps that provide a more holistic view of biostimulant effects under real-world conditions, in turn enabling industries and farmers to confidently and innovatively develop and successfully implement plant biostimulants. Thus, the study reported herein is a metabolomics work, involving use of artificial intelligence (AI)-driven strategies, to investigate the effects of microbial consortia on maize plants under field conditions. Exploring the emerging machine learning (ML) and deep learning (DL)-based methods for metabolite annotation, the maize metabolome under investigation could be comprehensively characterized. As such, this study aims to decode microbial consortia-induced metabolic reprogramming in maize plants under field conditions, revealing novel insights into the molecular mechanisms that drive the beneficial effects of biostimulants on plant health and growth. Generating such a hypothetical framework for modes of action of *Bacillus* consortia, the study sheds light on critical interactions between microbial biostimulants, plants, and their environment at molecular levels, providing actionable knowledge essential for advancing the biostimulant industry and shaping sustainable, effective agricultural practices across the sector.

## Experimental procedure

### Plant cultivation, treatments, and harvesting

In September and October 2019, maize (*Zea Mays* L.) was cultivated in an open field in KwaZulu-Natal, Dundee, South Africa (28°06′32.7″S 30°00′04.8″E), in sandy soil. During this period, Dundee’s subtropical highland climate brings average temperatures ranging from nighttime lows of about 12 °C to daytime highs of roughly 27 °C. The field trial included two treatments: a control group, termed also untreated control group (i.e., with no biostimulant application), and microbial biostimulant-treated groups (referred to as consortia 1, 2, and 3, and abbreviated as C1, C2, and C3, respectively). A statistical strip design with 12 paired strips was used and the field’s layout was set up to consist of two buffer zones on the outer regions, and the control and treated groups were alternating within the buffer regions. Due to the sizes of the fields, only two treatments could be applied per field area, thus two field areas were set up, each with its control and treatment groups: control 1 (CL1, the control group for consortium 2, C2) and control 2 (CL2, the control group for consortia 1 and 3, C1 and C3). Each biostimulant treatment (C1, C2, and C3) was formulated to a concentration of 2 billion colony-forming units per milliliter (CFU/mL). For each treatment, 1 L of the biostimulant was mixed with 82 L of water. This diluted solution was then applied to the soil furrow containing seeds for a 1-hectare area. Due to the Omnia trademark and commercialization, details regarding the preparation of the biostimulant formulation cannot be disclosed in this study. Notably these microbial consortia are *Bacillus*-based formulations: consortium 1 is a mixture of five *Bacillus* isolates, namely two *B. licheniformis* strains, two *B. laterosporus* strains, and one *B. amyloliquefaciens* strain; consortium 2 is a combination of *B. subtilis* strain, *B. pumilus* strain, and *B. amyloliquefaciens*; and consortium 3 is a formulation of two *B. licheniformis* strains, one *B. laterosporus* strain, and one* B. megaterium.*

The biological changes in the maize plants resulting from the applied treatments were observed over a period of 16 weeks after emergence (WAE). WAE indicates the number of weeks since the maize plants emerged. Maize leaves were harvested randomly from five different plants in each group (i.e., untreated control and biostimulant-treated plants) at three intervals: first at the vegetative stage (5 WAE), second at the vegetative/reproductive stage (9 WAE), and finally at the reproductive stage (16 WAE). To halt enzymatic activity and metabolic processes, the leaves were quickly cut and frozen in liquid nitrogen. The collected plant material was stored at − 20 °C until metabolite extraction.

### Metabolite extraction

Metabolites were extracted by first grinding the leaves into a fine powder using a marble pestle and mortar with liquid nitrogen. To avoid contamination and sample mix-up, the pestle and mortar were thoroughly washed with distilled water, followed by a rinse with 80% methanol between each sample. Two grams (2 g) of maize leaf powder was placed into a clean Falcon tube, and 20 mL of 80% cold methanol was added (1:10 m/v ratio). The mixture was sonicated for 1 min using a probe sonicator (Bandelin Sonopuls, Germany) set at 55% power. The sonicator probe was cleaned with 80% methanol between samples to prevent cross-contamination. The homogenates were centrifuged at 3750 rpm for 30 min at 4 °C, after which the supernatants were collected, and the pellets were discarded. The samples were then concentrated using a rotary evaporator (Büchi Rotavapor R-200), reducing the 20 mL homogenate to around 1 mL at 55 °C, and transferred to 2 mL Eppendorf tubes. A dry bath was used to completely evaporate the samples. The dried extracts were resuspended in 500 μL of a methanol:milliQ water mixture (1:1, *v*/*v*) and filtered through 0.22-μm nylon filters into pre-labeled HPLC glass vials with 500 μL inserts (Shimadzu, South Africa). The filtered samples were stored at 4 °C until LC–MS/MS analysis. In addition, quality control (QC) samples were prepared by pooling equal volumes from all the samples.

### Sample analysis on LC–MS/MS system

Sample analyses were performed using a Shimadzu LCMS-9030 qTOF liquid chromatography–quadrupole time-of-flight tandem MS (LC-qTOF-MS/MS) system (Shimadzu Corporation, Kyoto, Japan) equipped with an electrospray ionization (ESI) source. A Shim-pack Velox C18 column (100 mm × 2.1 mm × 2.7 µm) was used for chromatographic separation at a temperature of 50 °C, with a 5 µL injection volume. The mobile phase consisted of 0.1% aqueous formic acid (solvent A) and 0.1% formic acid in acetonitrile (solvent B). The gradient elution was performed at a steady flow rate of 0.4 mL/min. Solvent B started at 3% (*v*/*v*) for the first 3 min, then increased to 40% between 3 and 12 min, and further rose to 95% between 12 and 16 min. In the final phase of the run, from 18 to 20 min, the gradient returned to 3%. The total run time was 20 min, and each sample was analyzed in duplicate to account for variability.

The qTOF-high-definition mass spectrometer was set to acquire negative electrospray ionization data and was used to analyze the chromatographic effluents. ESI negative mode was selected based on preliminary experiments that indicated higher detection of ion peaks (data not presented). The instrument parameters were set as follows: an interface voltage of 4.0 kV, interface temperature at 300 °C, nebulization and dry gas flow rate at 3 L/min, heat block temperature of 400 °C, DL temperature at 280 °C, detector voltage at 1.8 kV, and flight tube temperature at 42 °C. To monitor and maintain high mass accuracy in the system, sodium iodide (NaI) was used as a calibration solution. Both MS^1^ and MS^2^ spectra were generated for ions within the m/z range of 100–1000 Da, with a minimum intensity threshold of 5000 counts. Data-dependent acquisition (DDA) was employed for fragmentation, using argon as the collision gas at a collision energy of 30 eV with a spread of 5 eV, to acquire MS^2^ spectra for all ions with the MS^1^-specified intensity threshold. Quality control (QC) samples were injected at both the start and end of each sample batch to ensure data validation and system stabilization. In addition, QC samples were analyzed every ten injections to monitor and address any changes in the instrument’s response.

### Molecular networking, metabolite annotation, and functional analysis

The raw MS/MS data from the LC–MS/MS analysis were converted to mzML format using MS converter software and processed with Mass Spectrometry-Data Independent Analysis (MS-DIAL) software. Data processing for all treatments followed these parameters: a mass accuracy tolerance of 0.5 Da for both MS1 and MS2, a minimum peak height of 1000 amplitude, and a mass slice width of 0.1 Dalton (Da) for peak detection, with a 0.5 sigma window value and a 0.05 MS/MS abundance cut-off used for data deconvolution. Under alignment settings, a retention time tolerance of 0.2 min was applied, using one of the QC samples as a reference file.

The resulting feature table, composed of Rt-*m/z* variable pairs and corresponding *m/z* peak intensities for each sample, was uploaded to SIMCA version 17.0 software (Sartorius, South Africa) for machine learning (ML) modeling. To ensure that the biological question of the study is accurately answered as competently as possible, various validations and data scrutiny were done. These included assessment of the number of extracted features (< 10,000 features, as a rule of thumb), applying the 80% rule (i.e., features found in < 20% of the analyzed samples were removed) and monitoring the quality of data and stability of the analysis using QC samples. Sample and feature normalization methods were employed to put all variables on equal footing, minimize variable redundancy, and adjust for measurement errors. Pareto-scaling was used as a variable normalization method as this provides a balance between emphasizing low-abundance features and minimizing the over-amplification of noise (van den Berg et al. 2006). A nonlinear iterative partial least squares algorithm (in-built within SIMCA) was used to handle the missing values, with a correction factor of 3.0 and a default threshold of 50%. Both unsupervised principal component analysis (PCA) and supervised (orthogonal) partial least squares discriminant analysis, (O)PLS-DA, models were generated, using a sevenfold cross-validation, expressed through metrics such as cumulative *R*^2^ (explained variance) and cumulative *Q*^2^ (predictive variance), and only the components positively contributing to increase the prediction ability of the model (*R1* significant components) were considered. For supervised models, additional validation included receiver operating characteristic (ROC) curves and permutation tests.

For metabolite annotation, AI-driven strategies, such as ML-based computational frameworks including molecular networking methods, were applied, enabling semi-automated annotation of spectral data. The mzML files were then uploaded to the CMN workflow on the GNPS ecosystem. The parameters used for generating CMN included a fragment ion mass tolerance of 0.5 Da and a precursor ion mass tolerance of 0.5 Da across all datasets. The cosine score threshold was set at 0.6, with a minimum of four matched fragment ions required for both datasets. Network visualization and analysis were performed using Cytoscape software (version 3.10.1) (Shannon 2003; Smoot et al. 2010). Furthermore, plantMASST (https://masst.gnps2.org/plantmasst/) was used to enhance annotation. MS/MS spectrum data, in the form of a universal spectrum identifier (USI), were searched in plantMASST. The default search parameters, including a cosine threshold of 0.7, a minimum of three matched peaks, and a precursor mass and fragment tolerance of 0.05 Da, were used. After initiating the search, the MS/MS spectrum was observed, along with an interactive taxonomic tree visualizing the results. Spectral libraries used include the MS-DIAL metabolomics MSP spectral kits (comprising all public MS/MS libraries), GNPS libraries queering mass spectral and structural databases such as CHEBI, DRUGBANK, GNPS, HMDB, and SUPNAT.

All matched and some unmatched/unidentified nodes visualized in Cytoscape were verified or tentatively annotated using their empirical formulae calculated from accurate mass and fragmentation patterns from MS2 analysis. Manual inspection of these annotations was complemented by searches in natural product dereplication databases, including PubChem (https://pubchem.ncbi.nlm.nih.gov/), Dictionary of Natural Products (http://dnp.chemnetbase.com/faces/chemical/ChemicalSearch.xhtml), and ChemSpider (https://www.chemspider.com/). The annotations were also verified and confirmed against available literature, against annotation details of metabolites in maize. The metabolites were annotated with confidence level 2, as defined by the Metabolomics Standard Initiative (MSI) (Sumner et al. 2007). All validated metabolites are presented in Table S1. The CMN results and putative metabolite annotations for this study are accessible through the GNPS CMN job link attached in the supplementary materials. Furthermore, pathway analysis of the annotated metabolites was carried out using metabolomics pathway analysis (MetPA), a module within the MetaboAnalyst bioinformatics tool suite (version 6.0; http://www.metaboanalyst.ca) (Pang et al. 2024). The annotated metabolites, identified with Kyoto Encyclopedia of Genes and Genomes (KEGG) (https://www.genome.jp/kegg/) identifiers, were uploaded into the Metabolomics Pathway Analysis (MetPA) tool, which uses the ‘relative betweenness centrality’ parameter and a hypergeometric test algorithm to identify, analyze, and visualize affected metabolic pathways.

## Results and discussion

### AI-driven characterization of maize metabolome

**Fig. 1:**
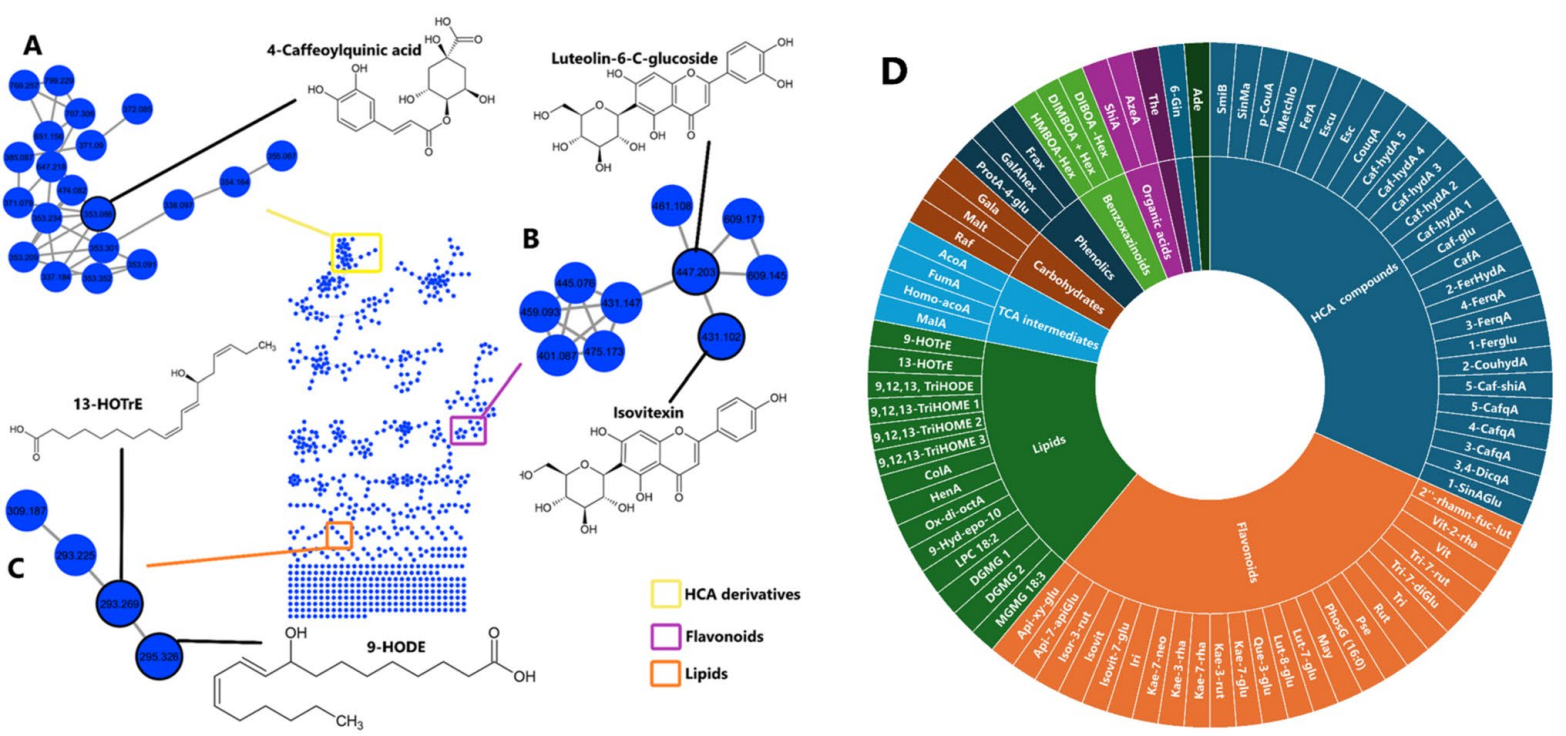
Classical molecular networking and metabolome coverage of maize leave extracts. The molecular network generated from maize leaf extracts treated with consortium 1 shows colored nodes, where those with black borders represent MS2 spectra matched to GNPS library spectra, and those without borders represent unmatched spectra. The zoomed-in clusters highlight major chemical classes, including **A** HCA derivatives (yellow), **B** flavonoids (purple), and **C** lipids (orange), with nodes labeled by their respective *m*/*z* values. Identified compounds include 4-caffeoylquinic acid (HCA derivatives), luteolin-6-C-glucoside and isovitexin (flavonoids), and 13-HotrE and 9-HODE (lipids). **D** The sunburst plot depicts globally annotated metabolites in maize leaf extracts and their assigned chemical classes, such as flavonoids, HCA derivatives, lipids, TCA intermediates, organic acids, amino acids, benzoxazinoids, amino acid derivatives, and nucleotides, with full metabolite names provided in Table S1 (colour figure online)

For semantics clarification and simplification, different expressions will be used interchangeably to refer to different experimental groups: for instance, expressions such as consortium 1, 2, 3 or consortia 1–3 or C1, C2, C3, will be used to refer to different groups of maize plants treated with different microbial consortia, i.e., consortium 1-treated group (C1), consortium 2-treated group (C2), and consortium 3-treated group (C3). As described in the experimental section, molecular networking (MN) method was performed to analyze and visualize the MS/MS data from the maize leave extracts. The MS-Cluster algorithm grouped ions detected within a specified mass tolerance into consensus spectra, which are then depicted as nodes. The generated molecular networks represent individual MS/MS spectra as nodes and edges represent relatedness in the MS/MS spectra (Quinn et al. 2017; Nothias et al. 2020). The constructed MN grouped spectral nodes into molecular families based on the GNPS spectral matching. For instance, maize treated with consortium 1 had 749 spectral nodes organized into 79 clusters (Fig. 1). On the other hand, maize treated with consortium 2 had 705 spectral nodes organized into 87 clusters (Fig. S1A). Lastly, maize treated with consortium 3 had 609 spectral nodes organized into 88 clusters (Fig. S1B).

A total of 342 spectral nodes across all treatment groups were matched and assigned metabolite names via GNPS library mass spectral databases, revealing distinct yet overlapping metabolomic signatures within the maize metabolome. Notably, upon careful examination and validation, flavonoids, hydroxycinnamic acids (HCAs), and lipids were the predominant metabolite classes among those identified (Fig. 1 and Table S1). As such, this computational strategy, organizing structurally related metabolites based on the spectral matching, provided insights into the chemical landscape of the maize plants treated with the different microbial consortia, under field conditions. To complement the MN outputs, the plant metabolite annotation and spectral search tool, plantMASST, was explored. The latter is a specialized computational tool and reference database designed for the analysis and identification of plant metabolites using mass spectrometry data (Gomes et al. 2024). In this study, unannotated or unmatched MS/MS spectra of the HCA cluster in the MN were searched directly against the plantMASST database, thus improving on the annotation. For instance, smilaside B and 3,4 dicaffeoylquinic acid were identified and confirmed using the plantMASST database (Figure S2).

Thus, using these AI-driven computational methods (molecular networking in GNPS and plantMASST), a total of 82 metabolites were annotated (Fig. 1D and Table S1). Flavonoids and hydroxycinnamic acids (HCAs) were major classes in the annotated maize metabolomic landscape (Fig. 1D). Flavonoids are a large class of secondary metabolites found in plants, characterized by their polyphenolic structure. Flavonoids also play a significant role in plant defenses against environmental stress (Mierziak et al. 2014; López 2019). HCAs, on the other hand, are a class of polyphenolic compounds primarily derived from the amino acid phenylalanine and are synthesized through the phenylpropanoid pathway. Like flavonoids, HCA derivatives play a defensive role in plants and are involved in plant growth regulation and signaling (Macoy et al. 2015; Liu et al. 2022). Furthermore, the other metabolites identified were mainly lipids (Fig. 1D). Lipids in plant cells have a wide variety of functions, both as structural components and bioactive substances (Suh et al. 2022). In the measured maize metabolome, several classes of metabolites, including flavonoids, HCA derivates, lipids, and benzoxazinoids, have been previously shown to play crucial roles in various biochemical and physiological processes throughout maize growth and development (Zhang et al. 2018; Butts-Wilmsmeyer et al. 2020; Gao et al. 2024; Zhao et al. 2024). To further understand the differential metabolic reprogramming in maize plants due to application of the three microbial consortia, under field conditions, both ML methods and functional analysis were then applied.

### Machine learning models: differentiating *Bacillus* consortia effects on maize metabolism

The principal component analysis (PCA) was applied to reduce data dimensionality and explore the data, revealing natural groupings and trends in the data sets (Jolliffe and Cadima 2016) (Fig. S3). The PCA score plot of the computed model (for all data, i.e., treatment groups and growth stages) revealed three main clusters, with some overlap between the consortia treatments and their respective controls (Fig. S3A), which were further revealed as corresponding to distinct growth stages of the maize (Fig. S3B). The first and second principal components (PC1 and PC2) explained 26.1% and 12.9% of the total variance, respectively (Fig. S3A, B). PC1 primarily reflected metabolomic reconfigurations associated with different plant growth stages in maize (Fig. 3A, B). In contrast, PC2 captured metabolic alterations related to the treatment effects, indicating treatment-induced metabolic reprogramming (Fig. 3A, B).

**Fig. 2:**
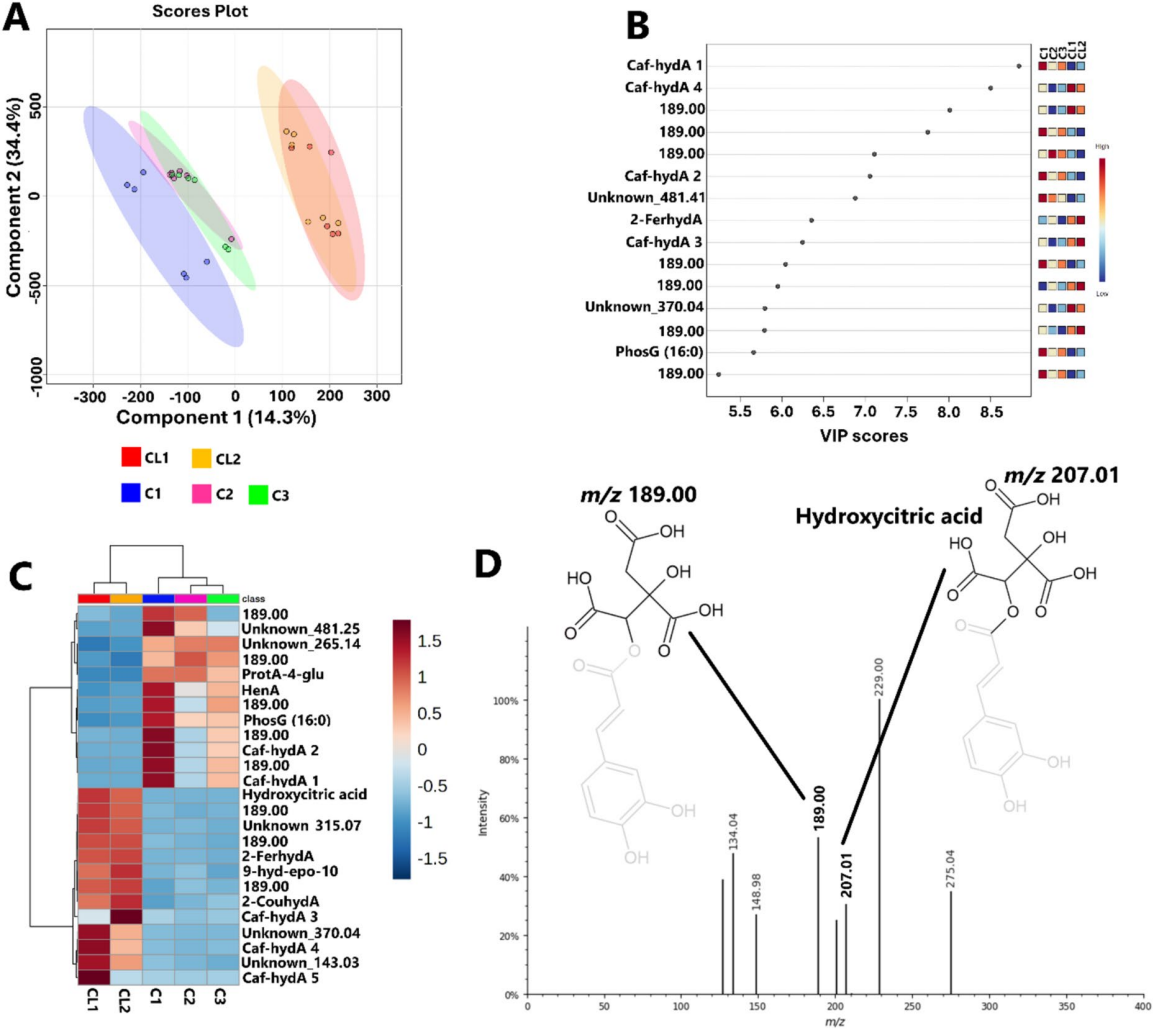
Supervised ML modeling, partial least squares discriminant analysis (PLS-DA), of maize data. **A** PLS-DA scores showing the grouping among the consortia treatment at the vegetative stage. **B** Variable importance projection (VIP) plots show the 15 discriminating variables (metabolites) that are responsible for the sample classification observed in (**A**) vegetative stage. **C** A complementary heat map showing the 25 variables (metabolites) contributing to the sample classification observed in the (**A**) vegetative stage. **D** Illustrating the fragmentation pattern of caffeoylhydroxycitric acid (precursor ion *m/z* 369.04, not shown in the spectrum). Full names of metabolites are provided in Table S1. *CL1/CL2* control, *C1* consortium 1-treated maize plants, *C2* consortium 2-treated maize plants, *C3* consortium 3-treated maize plants

To further investigate these metabolic alterations, a supervised ML method, PLS-DA, was applied. The computed PLS-DA model showed a separation between maize plants treated with consortium 1 and those that were treated with consortia 2 and 3 during the vegetative stage, while plants treated with consortia 2 and 3 had an overlap (Fig. 2A). The first and second principal components (PC1 and PC2) explained 34.4% and 14.3% of the total variance, respectively. This suggests that the metabolomic profiles of maize plants treated with consortium 1 are distinct from those of maize plants that were treated with consortia 2 and 3. In the transition and reproductive stages, PLS-DA modeling showed overlapping among C1, C2, and C3 (Figs. S4A and S5A). This suggests that metabolic profiles of the C1, C2, and C3 maize groups are not significantly distinct from each other at the transition and reproductive stages. To identify the metabolites responsible for the separation among C1, C2, and C3-treated maize groups, discriminant features were selected based on their variable importance in projection (VIP) scores. As a result, 15 metabolites were identified as key markers (i.e., the metabolites with a VIP > 5.0), and their relative quantities were assessed at different growth stages (Figs. 2B and S4B–S5B). In the vegetative stage, the metabolites that differentiate the consortia’s effects included isomers 1-4 of caffeoylhydroxycitric acid, 2-feruloylhydroxycitric acid, and phosphatidylglycerol (16:0/0:0). As shown in Figs. 2B, S4B, and S5B, the fragment *m/z* 189, originating from caffeoylhydroxycitric acid (Fig. 2D) and 2-feruloylhydroxycitric acid (Table S1), was observed more frequently. This indicates that these compounds are prominent discriminant metabolites at different growth stages.

Complementarily, the relative quantification of the discriminatory metabolites at the vegetative stage was performed (Fig. 2C). Isomers 1 and 2 of caffeoylhydroxycitric acid, phosphatidylglycerol (16:0/0:0), were observed in high levels in C1 (Fig. 2C). Isomer 3 of caffeoylhydroxycitric acid was found at higher levels in CL2, while isomer 4 was more abundant in CL1 (Fig. 2C). 2-Feruloylhydroxycitric acid was present at high levels in both CL1 and CL2 (Fig. 2C). As such, these results suggest that HCA derivatives are the key discriminatory metabolites among the consortia’s effects at the vegetative stage. HCA derivatives have been shown to play a crucial role in plant defense mechanisms, potentially enhancing resilience against environmental stressors (López de Felipe 2023). This defensive role is particularly important during the vegetative stage when maize plants are rapidly growing and are more vulnerable to environmental stressors.

Similar to the vegetative stage, most HCA derivatives were identified as key discriminatory metabolites at the transition stage (Fig. S4B). Heatmap analysis of discriminatory metabolites revealed that isomer 2 of caffeoylhydroxycitric acid was in high levels in C2, whereas isomer 3 was present in high levels in both CL1 and CL2. Moreover, isomers 4 and 5 were found at high levels in CL1 (Fig. S4C). 2″-*O*-⍺-l-Rhamnosyl-6-C-fucosyl-luteolin was found in high levels in C1, phosphatidylglycerol (16:0/0:0) on the other hand was found more prominent in C2 (Fig. S4C). HCA derivatives are crucial for the plant’s antioxidant defense system, particularly during the transition stage when plants are susceptible to oxidative stress due to environmental factors such as drought and UV radiation. The synthesis of HCA derivatives is upregulated in response to drought and UV radiation, which enhances the plant’s ability to scavenge reactive oxygen species (ROS) and mitigate oxidative damage (Piasecka et al. 2017, 2020).

The metabolites that discriminate C1, C2, and C3 in the reproductive stage included 4-caffeoylquinic acid and homoaconitic acid (Fig. S5B). Heatmap analysis showed that 4-caffeoylquinic acid was in high levels in C1, while homoaconitic acid was in high levels in the control group (CL1 and CL2) (Fig. S5C). As such, similar to vegetative and transition stage, HCA derivatives were identified as key discriminatory metabolites in the reproductive stage. HCA derivatives contribute to cell wall structure and rigidity. They are esterified with cell wall polysaccharides, lignin, and proteins, which enhance the mechanical strength of the plant (Nieter et al. 2015; Kaur and Goyal 2021; Feijao et al. 2022; Khawula et al. 2023). During the reproductive stage, maize undergoes pollination therefore, structural reinforcement is essential for supporting reproductive organs during pollination. Thus, these ML models, both PCA and PLS-DA, revealed differential metabolomic landscapes of maize plants treated with different *Bacillus* consortia, under field conditions. HCA derivatives were found to be the key metabolic markers that differentiated the effects of consortia treatment on maize plants at different growth phases.

**Fig. 3:**
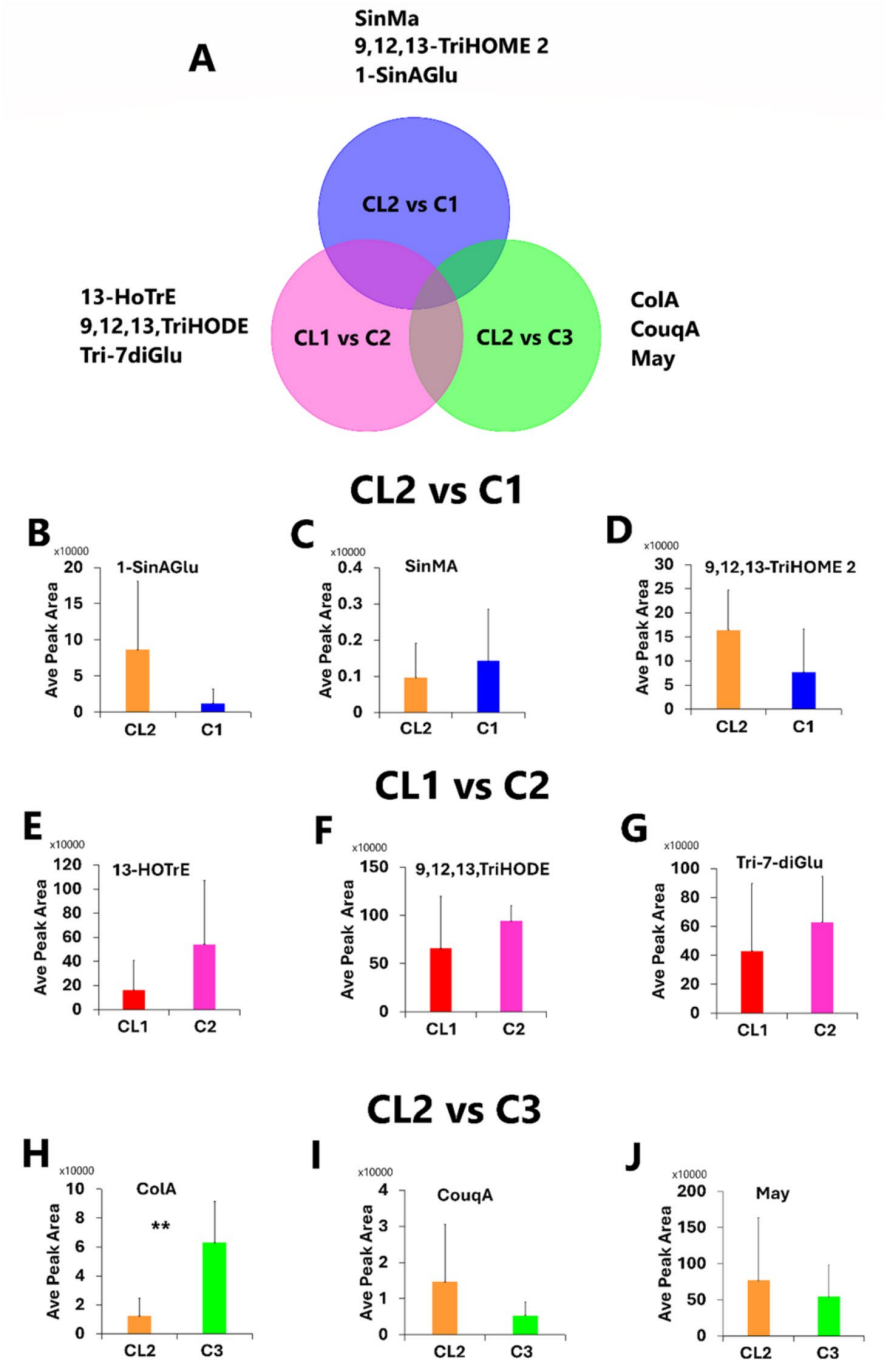
Unique putatively annotated metabolites extracted from orthogonal partial least squares discriminant analysis (OPLS-DA) S-plot and their relative quantification at the vegetative stage. **A** Venn diagram showing the unique metabolites identified as discriminatory metabolites between each consortium treatment and their control. **B**–**J** Quantification levels of unique discriminatory metabolite identified between each consortium treatment and their control. Refer to Table S1 for full names of metabolites. A *t* test was performed to assess the statistical significance of the comparisons between the control and treated groups. The error bars represent the standard deviations. The asterisks (**) indicate a *p* value ≤ 0.01, showing the significance of the differences between the control and consortia-treated group (CL2 vs C3), the *p* values of other comparisons were > 0.05 and are listed in Table S3. Full names of metabolites are provided in Table S1. *CL1/CL2* control, *C1* consortium treatment, *C2* consortium 2 treatment and *C3* consortium 3 treatment. *Ave* average

Building on the principles of PLS-DA, orthogonal partial least squares discriminant analysis (OPLS-DA) further refines this process by separating Y-predictive variation from Y-orthogonal variation. Focusing solely on the vegetative stage, OPLS-DA was applied, as a binary classification, to discriminate between untreated plants and those treated with consortia, aiming to extract key signatory metabolites that differentiate the effect of each consortium treatment on maize plants under field conditions (Figs. S4–S8). The OPLS-DA score plots displayed the classification of field maize samples, differentiating between control plants and those treated with consortia at the vegetative stage (Figs. S4A–S8A). The corresponding OPLS-DA loading S-plots revealed the distinctive metabolite features that separate the sample groupings shown in the score plots (Figs. S6B–S8B). The computed OPLS-DA models were validated using permutation tests (*n* = 100), *R*^2^ and *Q*^2^ metrics (Figs. S6C–8C), and receiver operator characteristic (ROC) curves (Figs. S6D–8D). The models demonstrated high predictability, outperforming permutated models, with ROC curves showing 100% sensitivity and specificity, thereby confirming their statistical reliability. While ROC analysis yielded 100% sensitivity and specificity, external validation is needed to confirm the model reliability. Furthermore, these models proved statistically reliable and outperformed the permutated models (*n* = 100) (Figs. S6D–S8D). Twenty-three metabolites were identified as discriminatory metabolites differentiating between the control group and consortia-treated maize groups at the vegetative stage (Table S2), and these are presented in a Venn diagram (Figs. 3A and S9). Herein, ‘unique’ refers to metabolites that were identified as discriminatory in one consortium-treated sample compared with the control condition but were not discriminatory in other consortium-treated samples and their respective controls.

Three metabolites were identified as unique metabolites that differentiate each consortium-treated group (C1, C2, and C3) from their respective controls (CL1 and CL2) (Fig. 3A). Furthermore, eight metabolites were identified as common discriminatory metabolites among the three consortia-treated groups and their respective controls (Fig. S9). C1 and C3 shared five identified metabolites as discriminatory metabolites that differentiate them from their respective control (CL2) (Fig. S9). In contrast, C2 and C3 had one common discriminatory metabolite identified that differentiated them from their respective controls (CL1 and CL2) (Fig. S9). Sinapoyl malate, 1-*o*-sinapoylglucose, and 9,12,13-TriHOME 2 were identified as unique discriminatory metabolites between CL2 and C1 (Fig. 3A). In contrast, 13-HOTrE, 9,12,13-TriHODE, and tricin 7-diglucuronoside were identified as unique discriminatory metabolites between CL1 and C2 (Fig. 3A). Colnelenic acid, coumaroyl quinic acid, and maysin, on the other hand, were identified as unique discriminatory metabolites between CL2 and C1 (Fig. 3A). These discriminatory metabolites are reflective of the plant’s developmental processes and its ability to interact with adverse environmental factors (Sun et al. 2015). This analysis helps in understanding how each consortium treatment affects the metabolic profile compared to the control, highlighting unique and shared metabolic responses. Thus, a quantitative analysis was performed to reveal the effect of the consortia treatment on the maize plants (Fig. 3B–J).

Consortium 1 application decreased the level of 1-*o*-sinapoylglucose while increasing the level of sinapoyl malate (Fig. 3B–D). 1-*O*-Sinapoylglucose, a glucose ester with a high free energy of hydrolysis, supplies the energy required for the transacylation reaction that produces sinapoyl malate in vegetative tissues (Lehfeldt et al. 2000; Bi et al. 2017; Fu et al. 2021; Nguyen et al. 2021). Thus, the decreased levels of 1-*O*-sinapoylglucose observed due to consortium 1 application suggest its utilization for the accumulation of sinapoyl malate, as shown in Fig. 3C. Sinapoyl malate absorbs UV-B radiation, thereby protecting plant tissues from potential damage caused by excessive UV exposure, which can lead to DNA damage and cell death (Mattana et al. 2005; Dean et al. 2014; Toldo et al. 2021). Studies have shown that sinapoyl malate accumulates in the upper epidermis of leaves, where it acts as a sunscreen, dissipating harmful UV radiation and preventing its penetration into deeper leaf tissues (Luo et al. 2017; Vink et al. 2023). This protective mechanism is particularly vital for maintaining the integrity of photosynthetic machinery and overall plant health under harmful UV radiation (Booker et al. 2012; Abiola et al. 2021). As such, it can be postulated that consortium 1 application activates the utilization of 1-*O*-sinapoylglucose for accumulation of sinapoyl malate which functionally enhances UV-B radiation tolerance during the vegetative stage at which the plant is more vulnerable to environmental stresses. Moreover, consortium 1 application decreased the levels of 9,12,13-TriHOME 2 in maize leaves. The mechanistic roles of the individual oxylipins are poorly understood. However, oxylipins are known to act as signaling molecules involved in regulating plant growth, development, and responses to both biotic and abiotic stresses (Offor et al. 2022; Sun et al. 2022).

13-HOTrE and 9,12,13-TriHODE were increased due to consortium 2 application (Fig. 3E, F). 13-HOTrE and 9,12,13-TriHODE are oxylipins; as highlighted above, the mechanistic roles of the individual oxylipins are poorly understood. However, the accumulation of oxylipins including 13-HOTrE has been shown to enhance the expression of defense-related genes, leading to a more robust immune response against pathogens (Adigun et al. 2023). As such, it can be postulated that the consortium 2 application enhances plant defense mechanisms through induced resistance. Moreover, an increase of flavonoid tricin 7-diglucuronoside was observed in consortium 2-treated maize plants. This observation is consistent with increased levels of tricin 7-diglucuronoside in plant growth-promoting rhizobacteria (PGPR)-treated plants compared to naïve stressed plants (Nephali et al. 2021). Moreover, the results suggested that PGPR treatment may enhance the accumulation of this flavonoid, which is associated with various biological functions in plants, including growth regulation and plant immunity. As such, it can be postulated that consortium 2 application enhances the plant’s immune response, stress tolerance, and growth regulation by promoting the accumulation of tricin 7-diglucuronoside.

Consortium 3 application increased the levels of colnelenic acid (Fig. 3H). Colnelenic acid is implicated in the regulation of plant hormone signaling pathways. For instance, Sanadhya et al. (2021) demonstrated that colnelenic acid and its related compounds are involved in activating defense responses against various pathogens. The study showed that the accumulation of colnelenic acid is associated with the activation of the 9-lipoxygenase (9-LOX) pathway, which is crucial for synthesizing oxylipins that function as signaling molecules in response to biotic stress. This suggests that consortium 3 application enhances the plant’s resistance to pathogens during vegetative stages by stimulating the production of colnelenic acid, thereby promoting healthier and more robust development. Moreover, consortium 3 application decreased the level of coumaroyl quinic acid. In a study by Comino et al. (2007), coumaroyl quinic acid was detected at low concentrations and is suggested to play a role as a transient intermediate in the biosynthesis of secondary metabolites, including flavonoids and lignin, as part of the broader phenylpropanoid pathway. As such, it can be postulated that consortium 3 application stimulated the use of coumaroyl quinic acid as a precursor for flavonoids and lignin biosynthesis which play a crucial role in plant defense mechanisms.

Eight metabolites were common discriminatory metabolites in the consortia-treated group and their respective controls (Fig. S9). These include 3-caffeoylquinic acid, 4-caffeoylquinic acid, 2-*O*-p-coumaroylhydroxycitric acid, 2-feruloylhydroxycitric acid, 2″-*O*-⍺-l-rhamnosyl-6-C-fucosyl-luteolin, heneicosanoic acid, 9-hydroxy-12,13-epoxy-10-octadecenoic acid, and aconitic acid (Fig. S9 and Table S2). These observations suggest that C1, C2, and C3 induce certain metabolic changes in maize plants similarly. Moreover, isomers 1–4 of caffeoylhydroxycitric acid and phosphatidylglycerol (16:0/0:0) were identified as common discriminatory metabolites in C1, C3, and their respective controls (Fig. S9 and Table S2). Caffeoylhydroxycitric acid has been shown to play a crucial role in plant defense mechanisms, potentially enhancing resilience against environmental stressors (Zhou et al. 2019). For instance, a study by Nephali et al., (2021) found that the accumulation of cinnamoyl hydroxycitric acid esters, which include caffeoylhydroxycitric acid, was observed in PGPR-treated plants under drought conditions. This suggests that these compounds may enhance drought resistance by providing defensive roles against environmental stresses. As such, it can be postulated that consortia 1 and 3 applications induce similar metabolic responses, possibly by promoting the accumulation of HCA derivatives. Lastly, apigenin-7-apioglucoside was the shared identified discriminatory metabolite between C2, C3, and their respective control (Fig. S9 and Table S2). Apigenin and its derivatives have been shown to have antifungal activity, which is crucial for protecting plants from various fungal infections (Fu et al. 2024). As such, it can be postulated that consortia 2 and 3 applications enhance the plant’s ability to defend against fungal pathogens by inducing the accumulation of apigenin-7-apioglucoside.

### Biostimulant-induced global metabolic reprogramming in maize plants

**Fig. 4:**
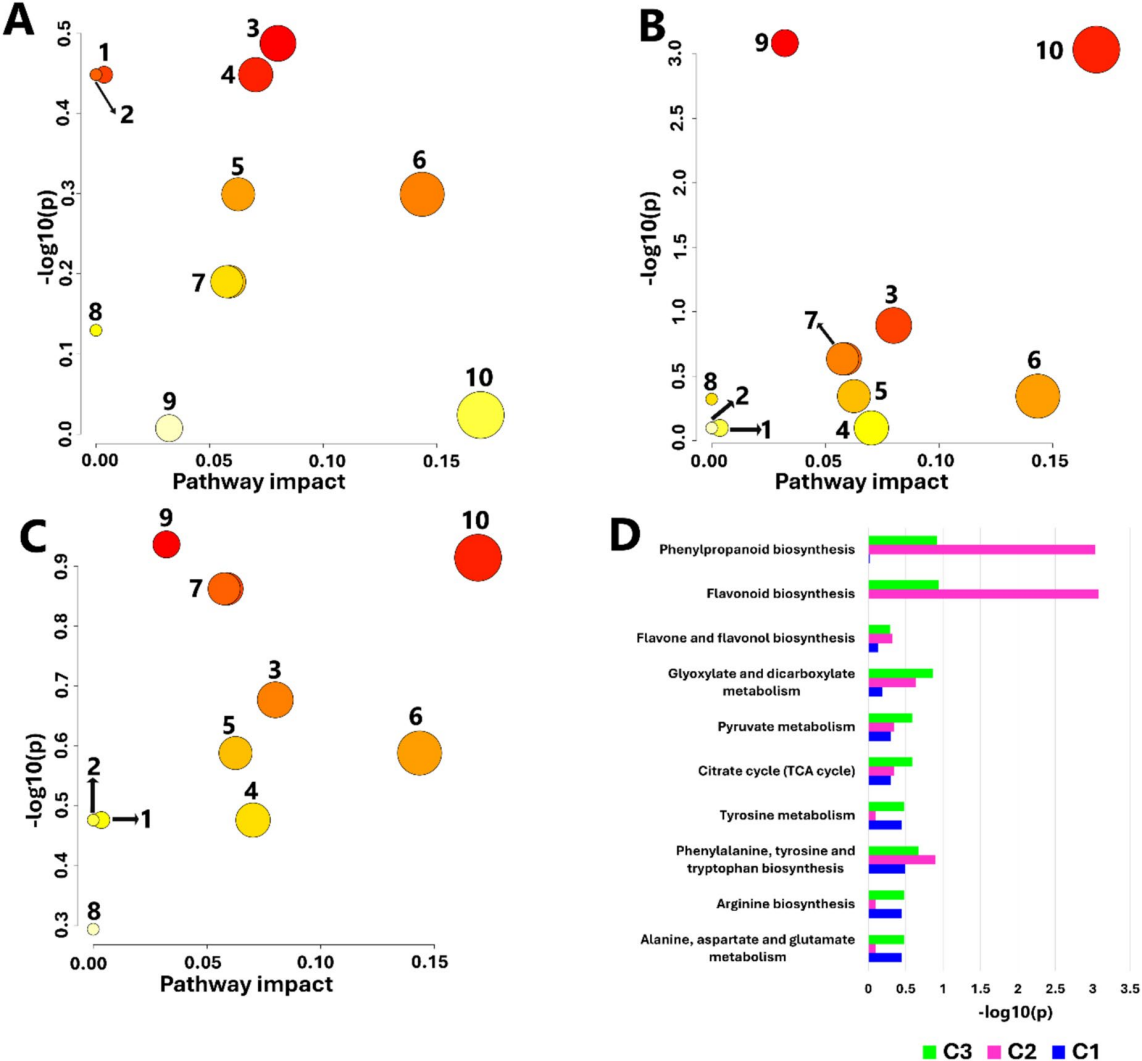
Metabolic pathway analysis at vegetative growth stage. A quantitative pathway analysis (QPA) showing all matched pathways according to the *p* values generated from the pathway enrichment analysis (*y*-axis) and pathway impact values calculated from pathway topology analysis (*x*-axis). **A** Represents pathway matched with maize-treated consortium 1, **B** maize treated with consortium 2, and **C** maize treated with consortium 3. The numbered pathways are (1) alanine, aspartate, and glutamate metabolism; (2) arginine biosynthesis; (3) phenylalanine, tyrosine, and tryptophan biosynthesis; (4) tyrosine metabolism; (5) the citrate cycle (TCA cycle); (6) pyruvate metabolism; (7) glyoxylate and dicarboxylate metabolism; (8) flavone and flavonol biosynthesis; (9) flavonoid biosynthesis; and (10) phenylpropanoid biosynthesis. The color of the nodes varies from light yellow to red, indicating the significance of each pathway based on the *p* value, with red representing the most significant. The size of the nodes reflects the impact of each pathway, with the largest nodes corresponding to those with the highest impact. **D** Bar graphs showing the -log10 (*p* value) of each pathway across different consortia treatments. *C1* maize treated with consortium1, *C2* maize treated with consortium 2, and *C3* maize treated with consortium 3 (colour figure online)

Both unique and shared metabolic changes in maize plants in response to treatment with the three different consortia (“Machine learning models: differentiating *Bacillus* consortia effects on maize metabolism” section) reflect a biostimulant-induced global metabolic reprogramming under field conditions. Quantitative pathway analysis (QPA) was employed to analyze and interpret these functional metabolic changes (Fig. 4A–C). A comparison of the metabolome view graphs for each treatment revealed that while pathway impact scores (*x*-axis) were consistent, the *p* values (vertical axis) varied between treatments (Fig. 4A–C). Thus, the *p* values for each pathway under different consortia applications were plotted in a bar graph to interpret the differences (Fig. 4D).

Bar graphs revealed the significantly altered biological pathways associated with three consortia applications in maize under field conditions included alanine, aspartate, and glutamate metabolism; arginine biosynthesis; phenylalanine, tyrosine, and tryptophan biosynthesis; tyrosine metabolism; the citrate cycle (TCA cycle); pyruvate metabolism; glyoxylate and dicarboxylate metabolism; flavone and flavonol biosynthesis; flavonoid biosynthesis; and phenylpropanoid biosynthesis (Fig. 4D and Table S4). Tyrosine metabolism was significantly altered due to consortia application (Fig. 4), and these findings are in concordance with previous findings where tyrosine metabolism was also significantly affected due to microbial biostimulant application (Lephatsi et al. 2022). Tyrosine metabolism is implicated in biological functions such as plant growth promotion. For instance, the former plays a crucial role in the synthesis of various essential metabolites, including tocopherols (vitamin E), plastoquinone, and ubiquinone. These compounds are crucial for plant growth and development, as they play roles in photosynthesis and cellular respiration (Xu et al. 2020). As shown in Fig. 4D, tyrosine metabolism was significantly impacted in maize treated with consortium 1 and 3 compared to consortium 2.

**Fig. 5:**
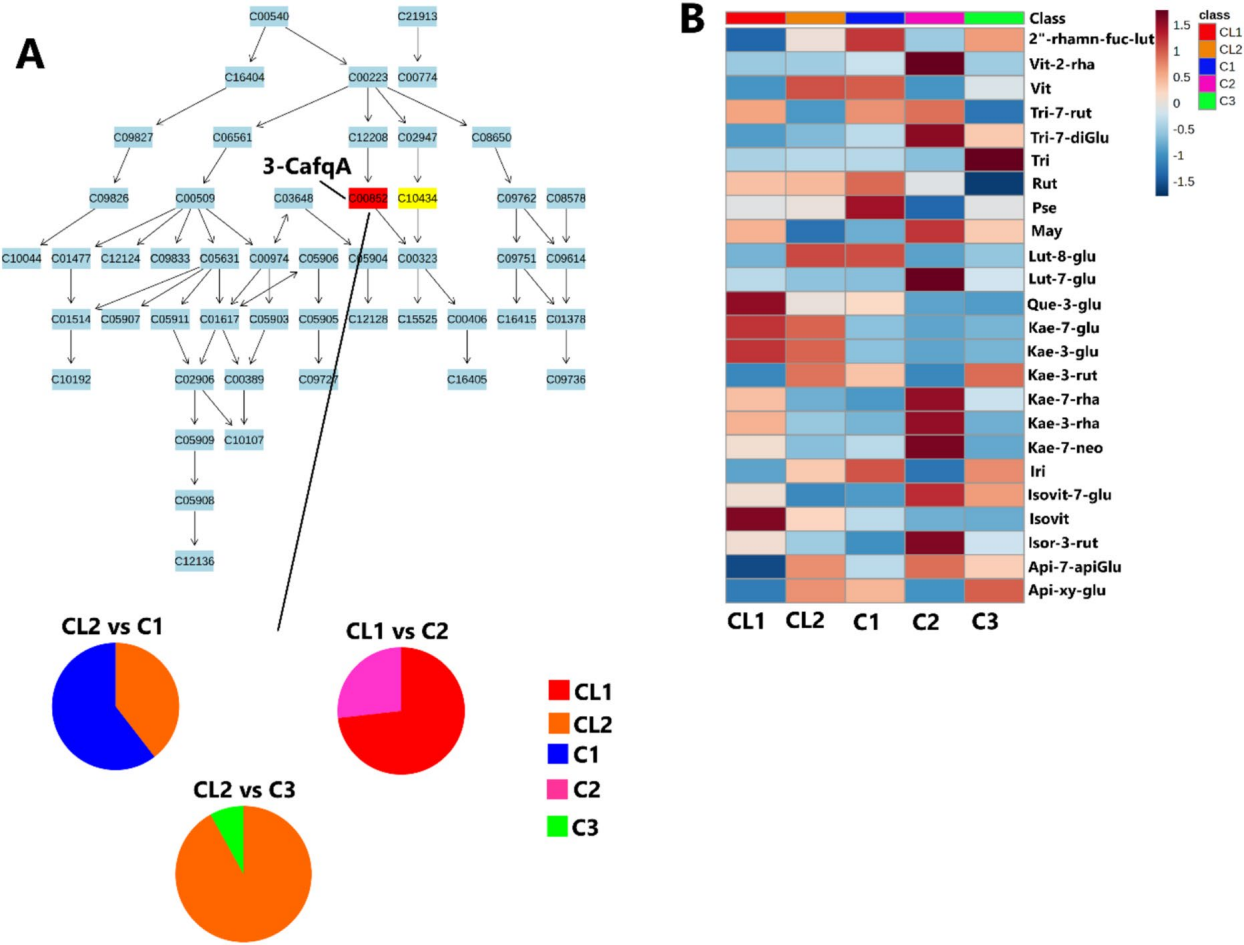
Metabolic pathway analysis and flavonoid relative quantification. **A** The topological pathway of flavonoid biosynthesis metabolism and quantification of matched metabolite (3-CafqA) within the pathway. **B** Heatmap showing relative quantification levels of flavonoids for control and consortia-treated samples at vegetative stage. Full names of metabolites are provided in Table S4.1. *CL1/CL2* control, *C1* maize treated with consortium 1, *C2* maize treated with consortium 2, and *C3* maize treated with consortium 3

Another altered pathway was the citrate cycle (TCA cycle) metabolism (Fig. 4). As previously mentioned during the vegetative stage, plants are primarily focused on growth, which requires significant energy. The TCA cycle facilitates the complete oxidation of acetyl-CoA to produce adenosine triphosphate (ATP), providing the necessary energy for processes such as cell division, elongation, and the synthesis of new tissues. This energy is crucial for the development of roots, stems, and leaves, which are essential for the plant’s overall growth and health (Wang et al. 2017). Correspondingly, citrate cycle metabolism was one of the pathways that were significantly impacted in the maize plants due to PGPR application (Nephali et al. 2021). Moreover, phenylalanine, tyrosine, and tryptophan biosynthesis pathway were significantly altered. Phenylalanine, tyrosine, and tryptophan are precursors for a wide range of secondary metabolites, such as flavonoids. These compounds are important for plant defense, pigmentation, and signaling (Tzin and Galili 2010; Dias et al. 2021; Shen et al. 2022). Similarly, phenylalanine, tyrosine, and tryptophan biosynthesis pathway was significantly impacted in maize plants due to microbial biostimulants (Lephatsi et al. 2022). The primary metabolism significantly impacted in this study such as alanine, aspartate, and glutamate metabolism; arginine biosynthesis; phenylalanine, tyrosine, and tryptophan biosynthesis; tyrosine metabolism; the citrate cycle (TCA cycle); pyruvate metabolism and glyoxylate and dicarboxylate metabolism encompasses essential biochemical processes that are crucial for plant growth and development (Rojas et al. 2014; Carrington et al. 2018; Pott et al. 2019; Zhou et al. 2024). As such, the application of these consortia has been shown to support the physiological events of the plant for growth promotion and enhanced defense. However, it is worth pointing out that the two highly impacted pathways are secondary metabolite metabolism, flavonoid biosynthesis, and phenylpropanoid biosynthesis (Fig. 4D). Consortium 2 application significantly altered these pathways compared to consortia 1 and 3 application (Fig. 4D). In the flavonoid biosynthesis pathway, 3-caffeoylquinic acid was mapped (Fig. 5A). In the phenylpropanoid metabolism pathway, 3-caffeoylquinic acid and ferulic acid were mapped (Fig. S10).

Phenolic compounds altered by the application of consortia in this study are flavonoids. To investigate the biochemical effects of the applied consortia on flavonoid levels during the vegetative stage, heatmaps were generated (Fig. 5B). Consortium 1 application increased the levels of 2″-*O*-⍺-l-rhamnosyl-6-C-fucosyl-luteolin, rutin, pseudobaptigenin, and iridin (Fig. 5B). In contrast, consortium 2 application elevated the levels of vitexin-2″-*O*-rhamnoside, tricin 7-diglucuronoside, maysin, luteolin-7-*O*-glucoside, kaempferol 7-*O*-rhamnoside, kaempferol 3-rhamnoside, kaempferol 7-neohesperidoside, isovitexin-7-*O*-glucoside, isorhamnetin 3-rutinoside, and apigenin-7-apioglucoside (Fig. 5B). Meanwhile, the application of consortium 3 increased the levels of tricin, kaempferol-3-*O*-rutinoside, and apigenin xyloside glucoside in maize plants (Fig. 5B). As previously mentioned during the early vegetative stages, maize plants are physiologically establishing a robust root system and developing initial leaf structures (Pongtip et al. 2024). Flavonoids serve as protective agents against abiotic stresses, including UV radiation. These metabolites absorb UV light, thereby shielding plant tissues from harmful radiation that can lead to cellular damage (Baskar et al. 2018; Shah and Smith 2020; Ferreyra et al. 2021).

Kaempferol glycosides are prevalent in certain plant tissues where they function as UV absorbers, protecting the plant against harmful UV rays (Dudek et al. 2016). For instance, Jan et al. (2022) found that kaempferol glycoside levels significantly increased in transgenic rice plants exposed to UV radiation stress, suggesting that this flavonoid plays a crucial role in the plant’s adaptive mechanisms to mitigate oxidative stress and enhance stress tolerance. This protective role is particularly important during the vegetative stage when plants are actively growing and are more susceptible to environmental stressors. Thus, the high levels of kaempferol glycosides observed due to the application of consortium 2 suggest that this treatment enhances stress tolerance and mitigates oxidative stress in the plants (Fig. 5B). In a study by Martin-Rivilla et al. (2020) conducted in greenhouses, microbial biostimulants significantly enhanced the flavonoid metabolism in blackberry leaves. The application of the microbial biostimulant led to an increase in the concentration of flavonoids, particularly kaempferol 3-*O*-rutinoside in treated plants compared to control plants. This increase was attributed to the activation of the flavonoid biosynthetic pathway, which is part of the plant’s induced systemic resistance (ISR) mechanism against biotic and abiotic stresses, including pathogen and oxidative stress. Therefore, the increased levels of kaempferol 3-*O*-rutinoside due to consortium 3 application may be involved in plant defense mechanism against pathogen and oxidative stress. Furthermore, flavonoids have been shown to influence auxin distribution, a key plant hormone that regulates growth patterns (Buer et al. 2010; Kumar and Pandey 2013). For instance, Peer et al. (2004) demonstrated that kaempferol glycosides play a crucial role in modulating auxin transport within *Arabidopsis thaliana*. Moreover, the authors showed that the presence of these flavonoids influences the localization and activity of PIN proteins, which are essential for the directional movement of auxin. This modulation of auxin transport subsequently affects root development, leading to alterations in root architecture and growth patterns (Peer et al. 2004).

**Fig. 6:**
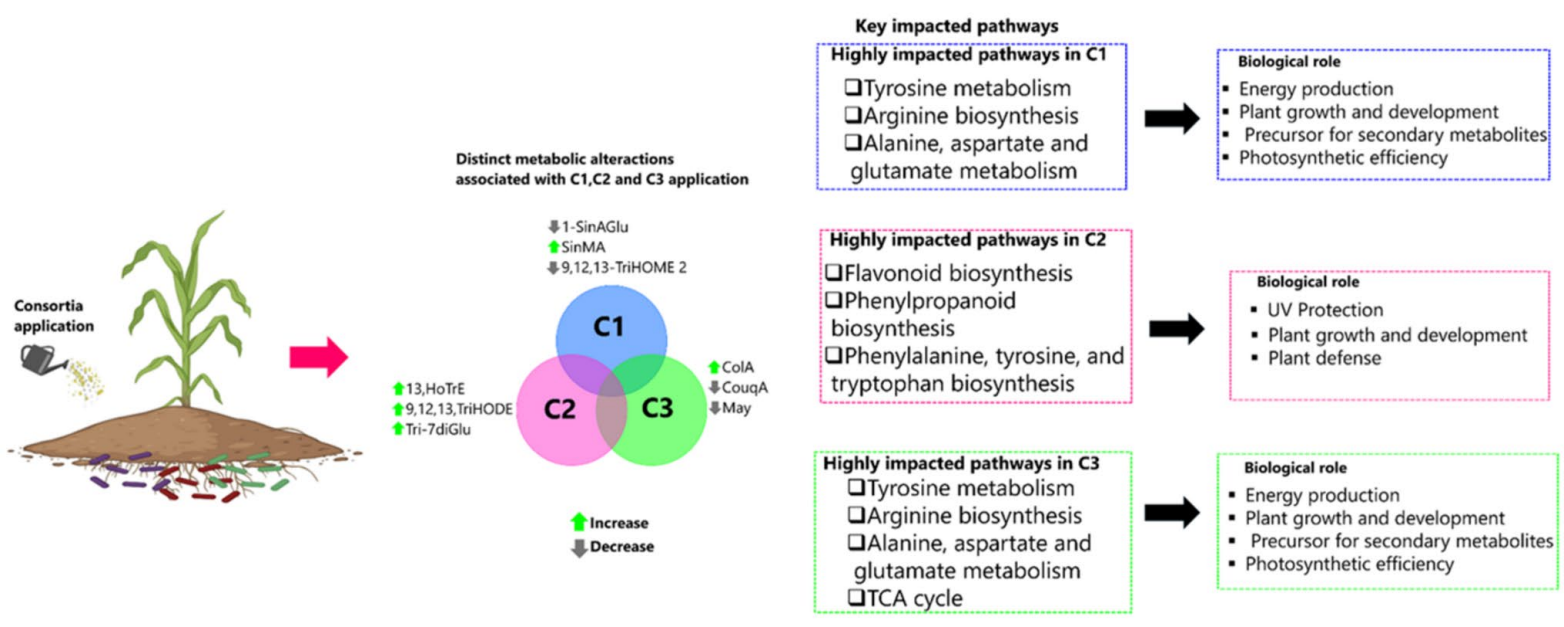
Summarized study’s findings highlight the key unique discriminatory metabolites between the consortia treatment and control and the impacted metabolic pathways due to consortia treatment. The left hand highlights the unique discriminatory metabolites identified between the consortia treatments and the control, showcasing the distinct metabolic changes induced by each consortium. The right hand illustrates the key pathways impacted by the consortia treatments, along with their associated biological roles. Furthermore, it highlights the highly impacted pathway resulting from the application of each consortium. Full names of metabolites are provided in Table S4.1. *CL1/CL2* control, i.e., plants with no biostimulant application, *C1* maize plants treated with consortium 1, *C2* maize plants treated with consortium 2, and *C3* maize plants treated with consortium 3

Thus, it can be postulated that the application of consortium 2 enhances plant defense mechanisms and development during the vegetative stage compared to other consortia. In summary, the application of consortia induced both common and unique metabolic changes in maize plants under field conditions. The unique metabolic changes were associated with distinct mechanisms that plants employed to defend themselves against stress. Consortia application influenced several key metabolic pathways, including arginine biosynthesis; phenylalanine, tyrosine, and tryptophan biosynthesis; tyrosine metabolism; the citrate cycle (TCA cycle); flavone and flavonol biosynthesis; flavonoid biosynthesis; and phenylpropanoid biosynthesis. Consortium 1 application highly altered alanine, aspartate, glutamate metabolism, arginine biosynthesis, and tyrosine metabolism (Fig. 6). Consortium 2 application, on the other hand, highly altered phenylalanine, tyrosine, and tryptophan biosynthesis, flavonoid biosynthesis, and phenylpropanoid biosynthesis (Fig. 6). Lastly consortium 3 application highly altered alanine, aspartate, and glutamate metabolism, arginine biosynthesis, tyrosine metabolism, and the citrate cycle (TCA cycle) (Fig. 6). Interestingly, applications of consortium 2 and consortium 3 significantly impacted similar metabolic pathways, despite differences in their formulation. These included alanine, aspartate, and glutamate metabolism; arginine biosynthesis; tyrosine metabolism; and the citrate cycle (TCA cycle) (Fig. 6). In contrast, applications of consortium 1 and consortium 3, which share *B. licheniformis* and *B. laterosporus*, showed more divergent plant responses, significantly impacting different metabolic pathways.

## Conclusion

This AI-integrated metabolomics reported herein intended to unravel the biochemical and molecular effects of three *Bacillus*-based microbial consortia on maize plants under real-world field conditions. By employing advanced computational strategies, including molecular networking, chemometrics, and supervised machine learning models, the study revealed distinct metabolomic trajectories induced by each consortium across key maize developmental stages. A central finding is the consistent identification of hydroxycinnamic acid (HCA) derivatives as discriminatory metabolites, suggesting their pivotal role in plant defense modulation and systemic metabolic reprogramming. The integration of artificial intelligence enabled high-resolution mapping of these metabolic networks and enabling deep functional insight. Importantly, the study proposes a novel mechanistic model of action for the three microbial consortia. Consortium 1 appears to operate via a structural and photoprotective mechanism, characterized by increased levels of compounds such as sinapoyl malate and a shift in HCA esterification, which may enhance UV-B tolerance and cell wall integrity. In contrast, consortium 2 engages a robust immuno-metabolic response, significantly upregulating flavonoid and phenylpropanoid biosynthetic pathways, including the accumulation of tricin 7-diglucuronoside and kaempferol glycosides, thereby promoting growth regulation, stress mitigation, and enhanced immune priming. Consortium 3 activates lipid-derived signaling pathways, notably increasing colnelenic acid and oxylipins, which are implicated in hormone-regulated defense mechanisms.

Collectively, these findings postulate a divergence model of the three biostimulants’ action: (i) photoprotection, structural reinforcement, and defense priming (C1), (ii) systemic metabolic priming for growth and defense (C2), and (iii) hormonal signaling modulation for stress response (C3). Thus, by conducting this work under agronomic field conditions and leveraging artificial intelligence across all analytical layers, the study bridges a critical gap between laboratory-based efficacy testing and practical field performance. It provides actionable mechanistic insights that can be harnessed to rationally design precision microbial biostimulants tailored to specific crop phenophases and stress environments. These insights represent a significant advancement for the biostimulant industry, offering a data-driven blueprint for the next generation of sustainable, effective, and field-ready bioformulations.

## Supplementary Information

Below is the link to the electronic supplementary material.

Supplementary file1 (DOCX 1623 KB)

## Data Availability

The spectral data presented in this study are made available on the supplementary files as job links and can also be found in an online repository at https://massive.ucsd.edu/MSV000093886.
