# Supplementary material for: AI-integrated metabolomics maps functional divergence of microbial consortia in field-grown maize
Source: Plant Cell Rep. 2025 Sep 11;44(10):211. doi: 10.1007/s00299-025-03600-z (PMC12426160; doi:10.1007/s00299-025-03600-z)
Supplement: Supplementary file 1 — Supplementary file1 (DOCX 1623 KB) [file 299_2025_3600_MOESM1_ESM.docx]

**Supplementary materials**

**AI-integrated metabolomics maps functional divergence of microbial consortia in field-grown maize**

**Musiwalo Samuel Mulaudzi^1^, Lerato Pertunia Nephali^1,2*^, Fidele Tugizimana^1,2^***

**
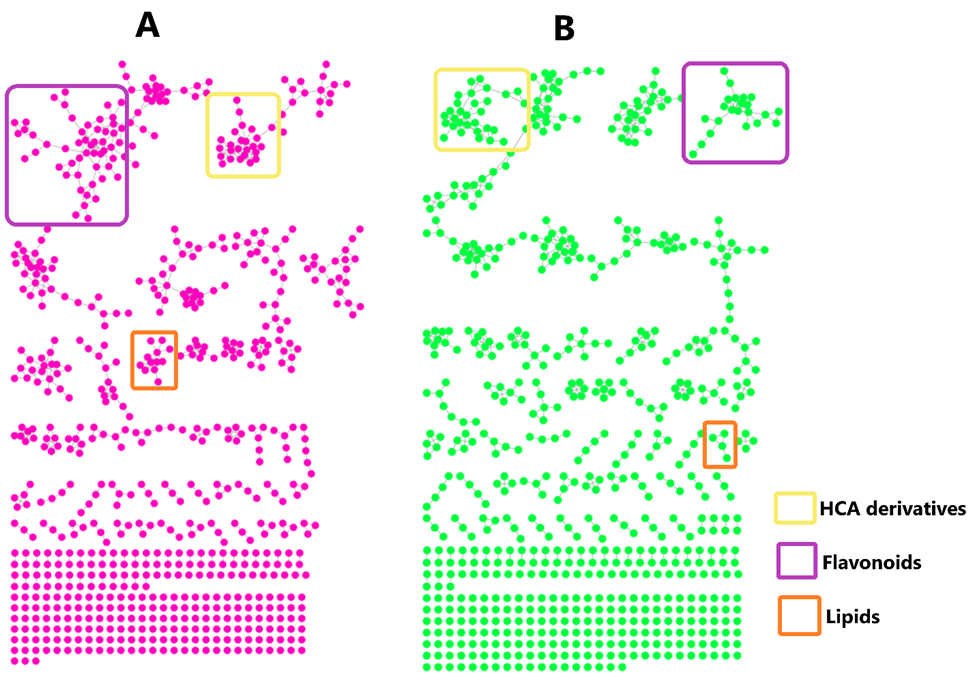
**

**Figure S1. Classical Molecular Networking**. Molecular network generated from maize leave extracts treated with consortia (**A**) 2 and (**B**) 3. Furthermore, it highlights some major chemical classes in maize leave extracts, such as HCA derivates (yellow), flavonoids (purple), and lipids (orange).


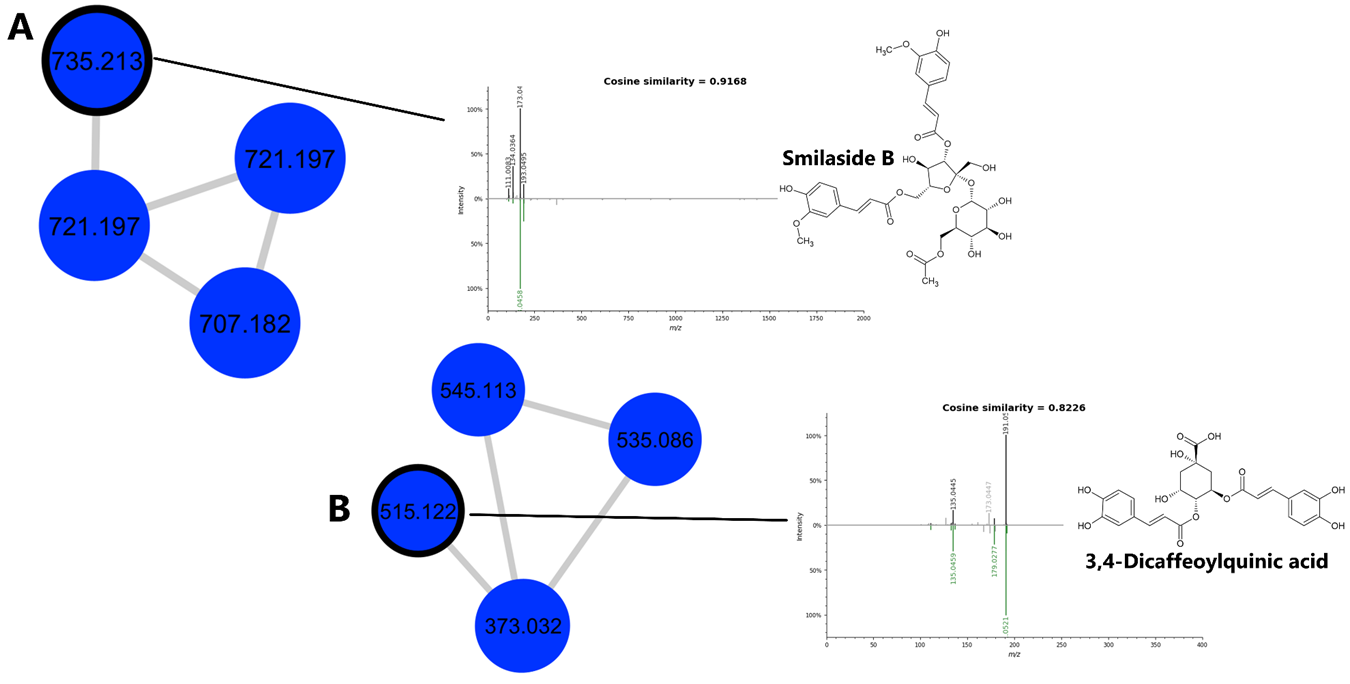


**Figure S2. plantMASST annotation.** Molecular network was generated with classical molecular networking, with spectra (nodes) searched against the plantMASST database. The plantMASST search output with matched nodes (**A**) Smilaside B and (**B**) 3,4-dicaffeoylquinic acid. Each matched compound is represented by its unique fragmentation pattern, confirming its identity through spectral alignment within the plantMASST database

**Table S1. Annotated metabolite table.** All putatively annotated metabolites from maize extracts based on the spectral matches of experimental data with the GNPS library databases and plantMASST.

| Putative annotation | Abbreviation | Molecular formula | Rt | *m/z* | Adduct | Fragments | Metabolite class |
| --- | --- | --- | --- | --- | --- | --- | --- |
| 1-o-sinapoylglucose | 1-SinAGlu | C_17_H_22_O_10_ | 6.22 | 385.11 | [M-H]- | 149,190,205,223,247, | HCA |
| 3,4-Dicaffeoylquinic acid | 3,4-DicqA | C_25_H_24_O_12_ | 5.91 | 515.12 | [M-H]- | 135,179 | HCA |
| 3-Caffeoylquinic acid | 3-CafqA | C_16_H_18_O_9_ | 5.22 | 353.08 | [M-H]- | 135,179,191 | HCA |
| 4-Caffeoylquinic acid | 4-CafqA | C_16_H_18_O_9_ | 3.05 | 353.08 | [M-H]- | 135,173,191 | HCA |
| 5-Caffeoylquinic acid | 5-CafqA | C_16_H_18_O_9_ | 4.89 | 353.09 | [M-H]- | 191 | HCA |
| 5-O-Caffeoylshikimic acid | 5-Caf-shiA | C_16_H_16_O_8_ | 5.94 | 335.07 | [M-H]- | 135,179 | HCA |
| 2-O-p-Coumaroylhydroxycitric  acid | 2-CouhydA | C_15_H_14_O_10_ | 5.65 | 353.05 | [M-H]- | 127,173,189 | HCA |
| 1-O-Feruloylglucose | 1-Ferglu | C_16_H_20_O_9_ | 6.07 | 355.10 | [M-H]- | 134,178,193 | HCA |
| 3-Feruloylquinic acid | 3-FerqA | C_17_H_20_O_9_ | 5.79 | 367.10 | [M-H]- | 134,193 | HCA |
| 4-Feruloylquinic acid | 4-FerqA | C_17_H_20_O_9_ | 8.45 | 367.10 | [M-H]- | 134,173,193 | HCA |
| 2-Feruloylhydroxycitric acid | 2-FerHydA | C_16_H_16_O_11_ | 6.06 | 383.06 | [M-H]- | 127,189 | HCA |
| Caffeic acid | CafA | C_9_H_8_O_4_ | 3.58 | 179.03 | [M-H]- | 135 | HCA |
| Caffeoylglucarate | Caf-glu | C_15_H_16_O_11_ | 1.73 | 371.11 | [M-H]- | 129,135,173,191,209 | HCA |
| Caffeoylhydroxycitric acid (Isomer 1) | Caf-hydA 1 | C_15_H_14_O_11_ | 3.44 | 369.04 | [M-H]- | 127,189, 207 | HCA |
| Caffeoylhydroxycitric acid (Isomer 2) | Caf-hydA 2 | C_15_H_14_O_11_ | 3.78 | 369.04 | [M-H]- | 127,189, 207 | HCA |
| Caffeoylhydroxycitric acid (Isomer 3) | Caf-hydA 3 | C_15_H_14_O_11_ | 4.33 | 369.04 | [M-H]- | 127,189, 207 | HCA |
| Caffeoylhydroxycitric acid (Isomer 4) | Caf-hydA 4 | C_15_H_14_O_11_ | 4.57 | 369.04 | [M-H]- | 127,189, 207 | HCA |
| Caffeoylhydroxycitric acid (Isomer 5) | Caf-hydA 5 | C_15_H_14_O_11_ | 4.73 | 369.04 | [M-H]- | 127,189, 207 | HCA |
| Coumaroyl quinic acid | CouqA | C_16_H_18_O_8_ | 5.15 | 337.09 | [M-H]- | 191 | HCA |
| Esculetin | Esc | C_9_H_6_O_4_ | 5.73 | 177.01 | [M-H]- | 105,133 | HCA |
| Esculin | Escu | C_15_H_16_O_9_ | 6.14 | 339.07 | [M-H]- | 105,133,161,177 | HCA |
| Ferulic acid | FerA | \| C_10_H_10_O_4_ \| \| --- \| | 5.79 | 193.04 | [M-H]- | 134 | HCA |
| Methyl chlorogenate | Metchlo | C_17_H_20_O_9_ | 6.26 | 367.10 | [M-H]- | 111,134,173,193 | HCA |
| p-Coumaric acid | p-CouA | C_9_H_8_O_3_ | 5.63 | 163.03 | [M-H]- | 119 | HCA |
| Sinapoyl malate | SinMa | C_15_H_16_O_9_ | 8.13 | 339.07 | [M-H]- | 121,223 | HCA |
| Smilaside B | SmiB | C_34_H_40_O_18_ | 6.27 | 735.21 | [M-H]- | 134,173,193 | HCA |
| Apigenin xyloside glucoside | Api-xy-glu | C_26_H_28_O_14_ | 6.46 | 563.14 | [M-H]- | 353,383, 443,447,473 | Flavonoids |
| Apigenin-7-apioglucoside | Api-7-apiGlu | C_26_H_28_O_14_ | 9.011 | 563.13 | [M-H]- | 269,563 | Flavonoids |
| Isorhamnetin 3-rutinoside | Isor-3-rut | C_28_H_32_O_16_ | 7.14 | 623.16 | [M-H]- | 299,300,314,315 | Flavonoids |
| Isovitexin | Isovit | \| C_21_H_20_O_10_ \| \| --- \| | 6.74 | 431.14 | [M-H]- | 311 | Flavonoids |
| Isovitexin-7-O-glucoside | Isovit-7-glu | C_27_H_30_O_15_ | 6.15 | 609.14 | [M-H]- | 311,341 | Flavonoids |
| Iridin | Iri | C_24_H_26_O_13_ | 8.04 | 521.13 | [M-H]- | 297,343,359 | Flavonoids |
| Kaempferol 7-neohesperidoside | Kae-7-neo | C_27_H_30_O_15_ | 7.19 | 593.15 | [M-H]- | 285 | Flavonoids |
| Kaempferol 3-rhamnoside | Kae-3-rha | C_21_H_20_O_10_ | 6.96 | 431.09 | [M-H]- | 285 | Flavonoids |
| Kaempferol 7-O-rhamnoside | Kae-7-rha | C_27_H_30_O_15_ | 7.41 | 431.09 | [M-H]- | 107,151,285,431 | Flavonoids |
| Kaempferol-3-O-rutinoside | Kae-3-rut | C_27_H_30_O_15_ | 7.35 | 593.14 | [M-H]- | 285 | Flavonoids |
| Kaempferol-7-O-glucoside | Kae-7-glu | C_21_H_19_O_11_ | 6.16 | 447.09 | [M-H]- | 447 | Flavonoids |
| Quercetin-3-O-glucoside | Que-3-glu | C_21_H_20_O_12_ | 6.71 | 463.08 | [M-H]- | 255,271,300,301 | Flavonoids |
| Luteolin-8-glucoside | Lut-8-glu | C_21_H_20_O_11_ | 7.11 | 447.21 | [M-H]- | 327 | Flavonoids |
| Luteolin-7-O-glucoside | Lut-7-glu | \| C_21_H_20_O_11_ \| \| --- \| | 6.36 | 447.09 | [M-H]- | 285,447 | Flavonoids |
| Maysin | May | C_27_H_28_O_14_ | 7.36 | 575.13 | [M-H]- | 337,411,473 | Flavonoids |
| Pseudobaptigenin | Pse | C_16_H_10_O_5_ | 7.736 | 281.04 | [M-H]- | 281 | Flavonoids |
| Rutin | Rut | C_27_H_30_O_16_ | 7.14 | 609.14 | [M-H]- | 293, 300,301 | Flavonoids |
| Tricin | Tri | C_17_H_14_O_7_ | 8.65 | 329.06 | [M-H]- | 299,314,315 | Flavonoids |
| Tricin 7-diglucuronoside | Tri-7-diGlu | C_29_H_30_O_19_ | 7.01 | 681.13 | [M-H]- | 131,175,351 | Flavonoids |
| Tricin 7-rutinoside | Tri-7-rut | C_29_H_34_O_16_ | 7.74 | 637.25 | [M-H]- | 329 | Flavonoids |
| Vitexin | Vit | C_21_H_20_O_10_ | 6.87 | 431.09 | [M-H]- | 117,283,311,341 | Flavonoids |
| Vitexin-2''-O-rhamnoside | Vit-2-rha | C_27_H_30_O_14_ | 6.70 | 577.15 | [M-H]- | 293,311,413 | Flavonoids |
| 2''-O-⍺-L-rhamnosyl-6-C-fucosyl-luteolin | 2''-rhamn-fuc-lut | C_27_H_30_O_14_ | 5.22 | 577.26 | [M-H]- | 164,225,299 | Flavonoids |
| 9-HOTrE | 9-HOTrE | C_18_H_30_O_3_ | 10.68 | 293.21 | [M-H]- | 121,171,231,275 | Lipids |
| 13-HOTrE | 13-HOTrE | C_18_H_30_O_3_ | 10.76 | 293.21 | [M-H]- | 195,223,293 | Lipids |
| 9,12,13, TriHODE | 9,12,13, TriHODE | C_18_H_32_O_5_ | 8.65 | 327.21 | [M-H]- | 171,211,327 | Lipids |
| 9,12,13-TriHOME 1 | 9,12,13-TriHOME 1 | C_18_H_34_O_5_ | 9.32 | 329.23 | [M-H]- | 139,181,182,197 | Lipids |
| 9,12,13-TriHOME 2 | 9,12,13-TriHOME 2 | C_18_H_34_O_5_ | 8.92 | 329.23 | [M-H]- | 139,182,200 | Lipids |
| 9,12,13-TriHOME 3 | 9,12,13-TriHOME 3 | C_18_H_34_O_5_ | 9.58 | 329.23 | [M-H]- | 139,181,182,197 | Lipids |
| Colnelenic acid | ColA | C_18_H_28_O_3_ | 11.57 | 291.19 | [M-H]- | 185, 253,277 | Lipids |
| Heneicosanoic acid | HenA | C_21_H_42_O_2_ | 11.49 | 325.18 | [M-H]- | 119,183 | Lipids |
| Oxo-dihydroxy-octadecadienoic  acid | Ox-di-octA | C_18_H_30_O_5_ | 9.66 | 325.20 | [M-H]- | 119,183 | Lipids |
| 9-Hydroxy-12,13-epoxy-10-  octadecenoic acid | 9-Hyd-epo-10 | C_18_H_32_O_4_ | 10.96 | 311.16 | [M-H]- | 170, 183,197 | Lipids |
| LPC 18:2 | LPC 18:2 | C_26_H_50_NO_7_P | 11.51 | 564.33 | [M+HCOO]- | 279 | Lipids |
| DGMG 18:3 1 | DGMG 1 | C_33_H_56_O_14_ | 10.88 | 721.36 | [M+HCOO]- | 311,397,415,577,675 | Lipids |
| DGMG 18:3 2 | DGMG 2 | C_33_H_56_O_14_ | 11.08 | 721.36 | [M+HCOO]- | 325,397,415,577,675 | Lipids |
| MGMG 18:3 | MGMG 18:3 | C_27_H_46_O_9_ | 11.18 | 559.31 | [M+HCOO]- | 2753,277,291,405,476 | Lipids |
| Phosphatidylglycerol (16:0/0:0) | PhosG (16:0) | C_42_H_77_O_10_P | 11.54 | 483.27 | [M-H]- | 153,227,245,255, 245 | Lipids |
| Aconitic acid | AcoA | C_6_H_6_O_6_ | 0.974 | 173.00 | [M-H]- | 85,111,129 | TCA |
| Fumaric acid | FumA | C_4_H_4_O_4_ | 0.80 | 115.00 | [M-H]- | 111 | TCA |
| Homoaconitic acid | Homo-acoA | C_7_H_8_O_6_ | 1.86 | 187.02 | [M-H]- | 85,111,129,143 | TCA |
| Malic acid | MalA | C_4_H_6_O_5_ | 0.80 | 133.01 | [M-H]- | 115 | TCA |
| Azelaic acid | AzeA | C_9_H_16_O_4_ | 7.15 | 187.09 | [M-H]- | 125 | Organic acids |
| Shikimic acid | ShiA | C_7_H_10_O_5_ | 6.26 | 173.14 | [M-H]- | 155 | Organic acids |
| DIBOA + O-Hex | DIBOA -Hex | C_14_H_17_NO_9_ | 2.62 | 342.08 | [M-H]- | 180 | Benzoxazinoids |
| DIMBOA + O-Hex | DIMBOA + Hex | C_15_H_19_NO_10_ | 6.17 | 372.21 | [M-H]- | 149,164 | Benzoxazinoids |
| HMBOA + O-Hex | HMBOA-Hex | C_15_H_19_NO_9_ | 6.12 | 356.09 | [M-H]- | 166, 194 | Benzoxazinoids |
| Fraxin | Frax | C_16_H_18_O_10_ | 5.53 | 369.08 | [M-H]- | 191 | Phenolics |
| Gallic acid hexoside | GalAhex | C_13_H_16_O_10_ | 1.41 | 331.09 | [M-H]- | 125,168 | Phenolics |
| Protocatechuic acid 4-glucoside | ProtA-4-glu | C_13_H_16_O_9_ | 1.98 | 315.13 | [M-H]- | 108,109,152,153 | Phenolics |
| Galactinol | Gala | C_12_H_22_O_11_ | 0.75 | 341.10 | [M-H]- | 101,179 | Carbohydrates |
| Maltotriose | Malt | C_18_H_32_O_16_ | 0.66 | 502.91 | [M-H]- | 161,172,179 | Carbohydrates |
| Raffinose | Raf | C_18_H_32_O_16_ | 2.31 | 503.13 | [M-H]- | 179 | Carbohydrates |
| Adenine | Ade | C_5_H_5_N_5_ | 1.28 | 134.04 | [M-H]- | 134 | Nucleotides |
| 6-Gingerol structural analog | 6-Gin | C_17_H_26_O_4_ | 9.04 | 293.17 | [M-H]- | 175 | Phenylpropanoid |
| Theanine | The | C_7_H_14_N_2_O_3_ | 6.69 | 173.09 | [M-H]- | 173 | Amino acid derivate |


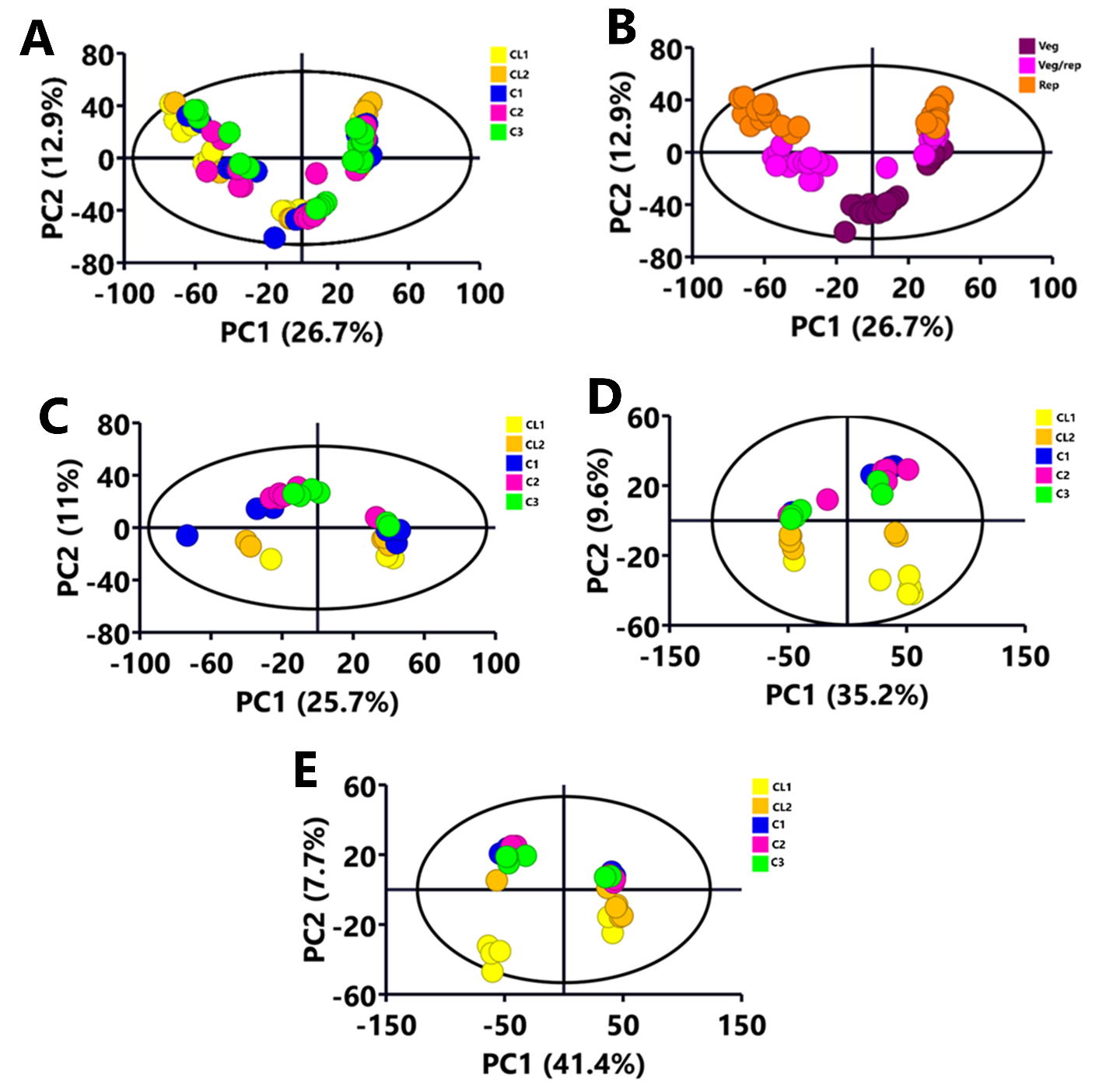


**Figure S3. Unsupervised chemometric modeling principal component analysis (PCA)**. Models include PCA score plots for (**A** and **B**) 8-component model, explaining 58.7% of the total variation in Pareto-scaled X data with a predictive power of 46.5%. The PCA score plot (**A**) shows clustering and grouping between treated and non-treated samples, and (**B**) shows clustering of samples based on maize growth stages. (**C**) 4-component model, explaining 50.1% of the total variation in Pareto-scaled X data with a predictive power of 20.3%, and (**D**) 4-component model, explaining 56.7% of the total variation in Pareto-scaled X data with the predictive power of 41.5%. (**E**) 4-component model, explaining 54.9% of the total variation in Pareto-scaled X data with a predictive power of 41.3%. The PCA score plots show the grouping between treated and non-treated samples based on maize growth stages, with (**A**) representing the vegetative stage, (**B**) representing the transition stage, and (**C**) representing the reproductive stage. **Abbreviations**: CL1/CL2 = control, C1= consortium 1 treatment, C2= consortium 2 treatment, C3= consortium 3 treatment, Veg= vegetative stage, Veg/rep= vegetative/reproductive (transition) stage, and Rep=reproductive stage.


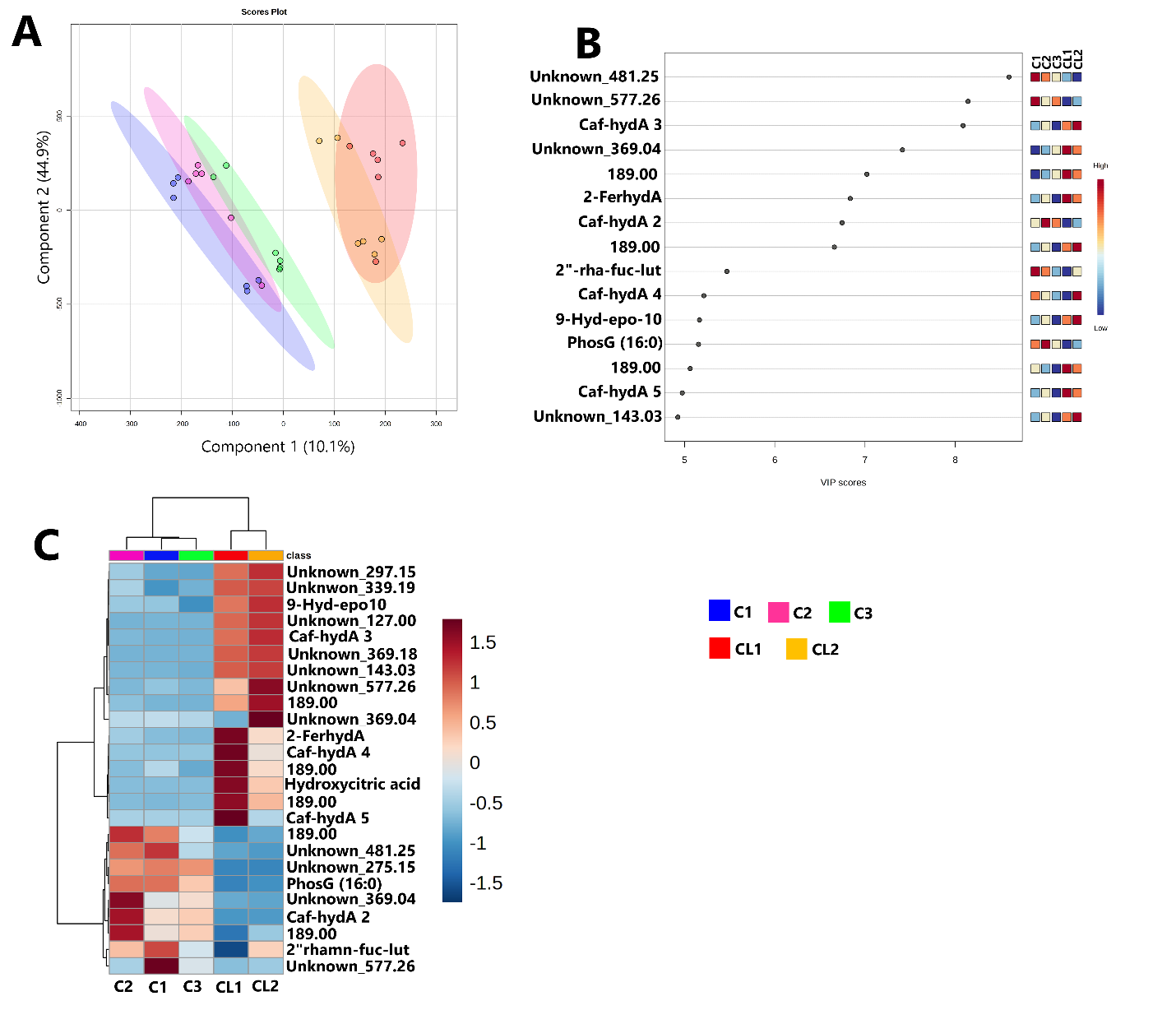


**Figure S4. Supervised chemometric modeling partial least squares discriminant analysis (PLS-DA) of maize extracts. (A)** PLS-DA scores showing the grouping among the consortia treatment at the transition stage. (**B**) Variable importance projection (VIP) plots show the 15 discriminating variables (metabolites) that are responsible for the sample classification observed in (**A**) transition stage. (**C**) Complementary heatmap showing the 25 variables (metabolites) contributing to the sample classification observed in the (**A**) transition stage. Full names of metabolites are provided in **Table S1. Abbreviations:** CL1/CL2 = control, C1= consortium 1 treatment, C2= consortium 2 treatment, C3= consortium 3 treatment.


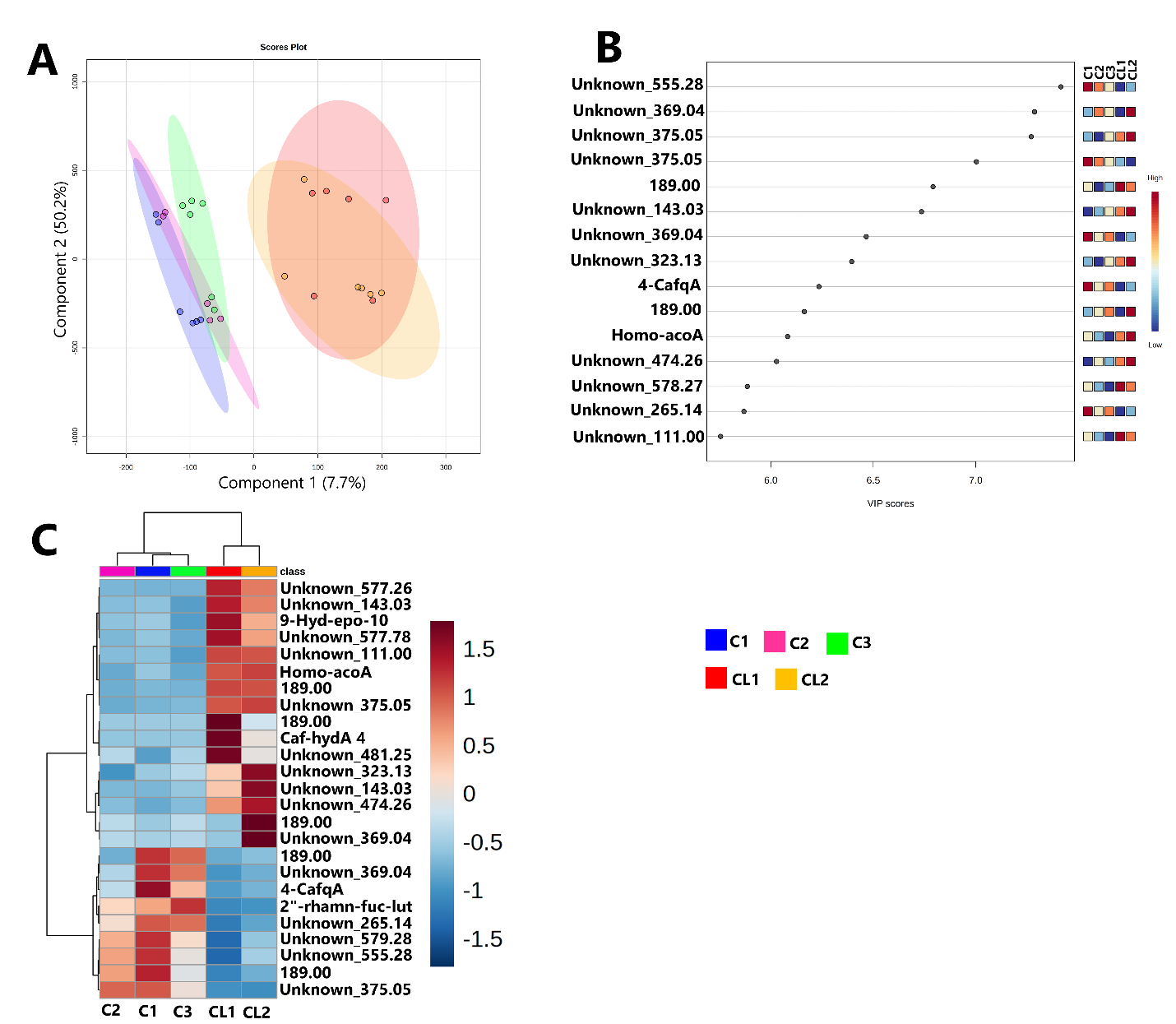


**Figure S5. Supervised chemometric modeling partial least squares discriminant analysis (PLS-DA) of maize extracts. (A)** PLS-DA scores showing the grouping among the consortia treatment at the reproductive stage. (**B**) Variable importance projection (VIP) plots show the 15 discriminating variables (metabolites) that are responsible for the sample classification observed in (**A**) reproductive stage. (**C**) Complementary heatmap showing the 25 variables (metabolites) contributing to the sample classification observed in the (**A**) reproductive stage. Full names of metabolites are provided in **Table S1.  Abbreviations:** CL1/CL2 = control, C1= consortium 1 treatment, C2= consortium 2 treatment, C3= consortium 3 treatment


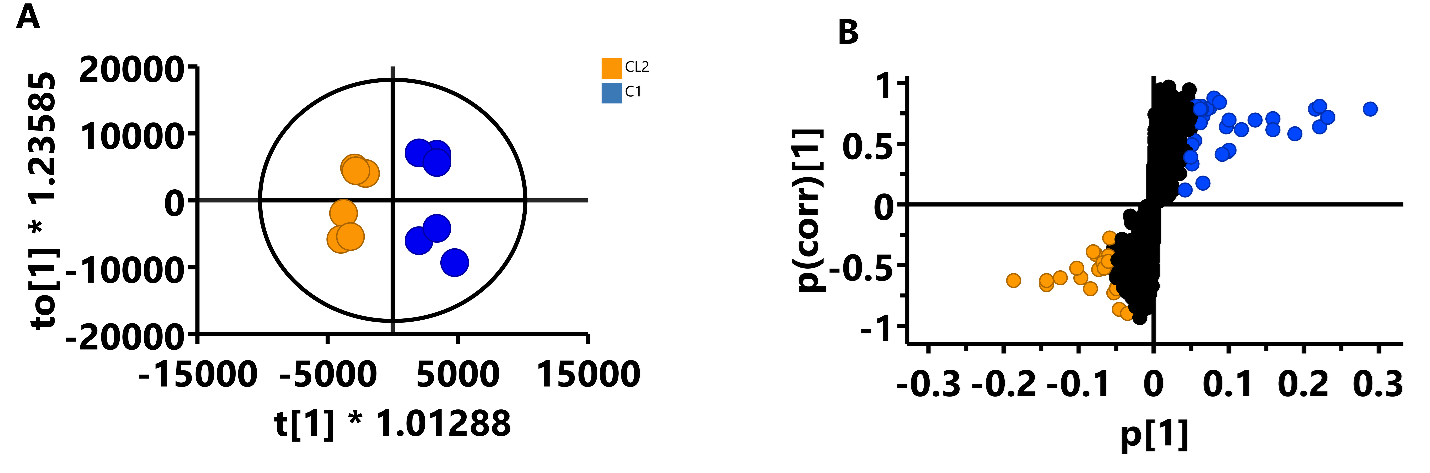


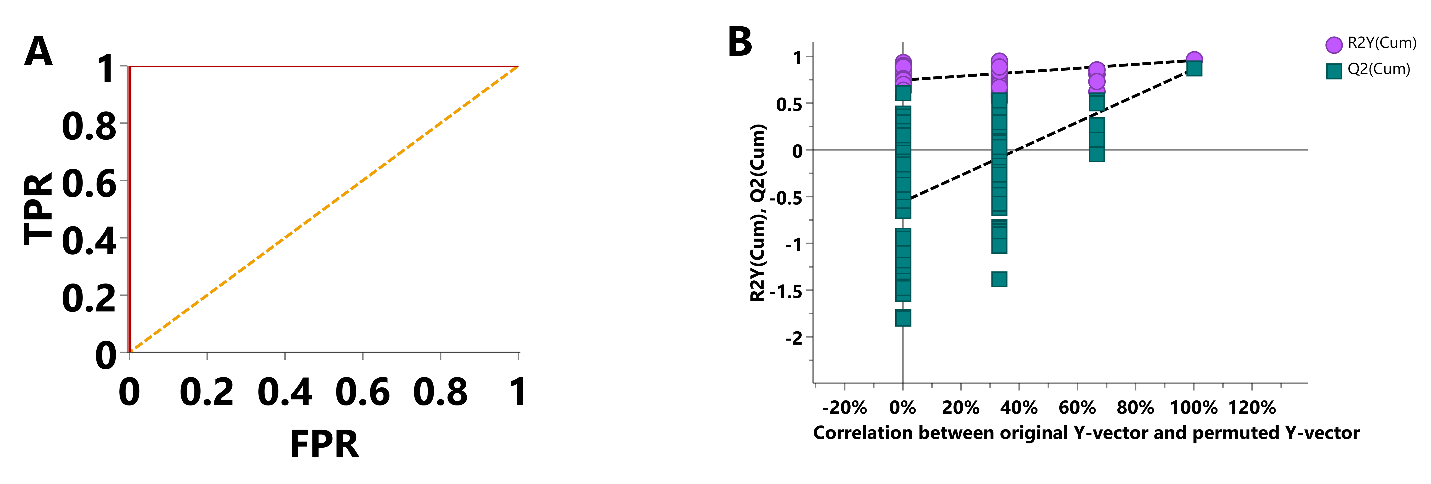
**Figure S6. Orthogonal partial least squares-discriminant analysis (OPLS-DA) validation for control vs consortium 1 application**. (**A**) Receiver operator characteristic (ROC) plot summarising the performance of the binary classifier. (**B**) Permutation test plot (n = 100) validating the predictive capability of the OPLS-DA model.


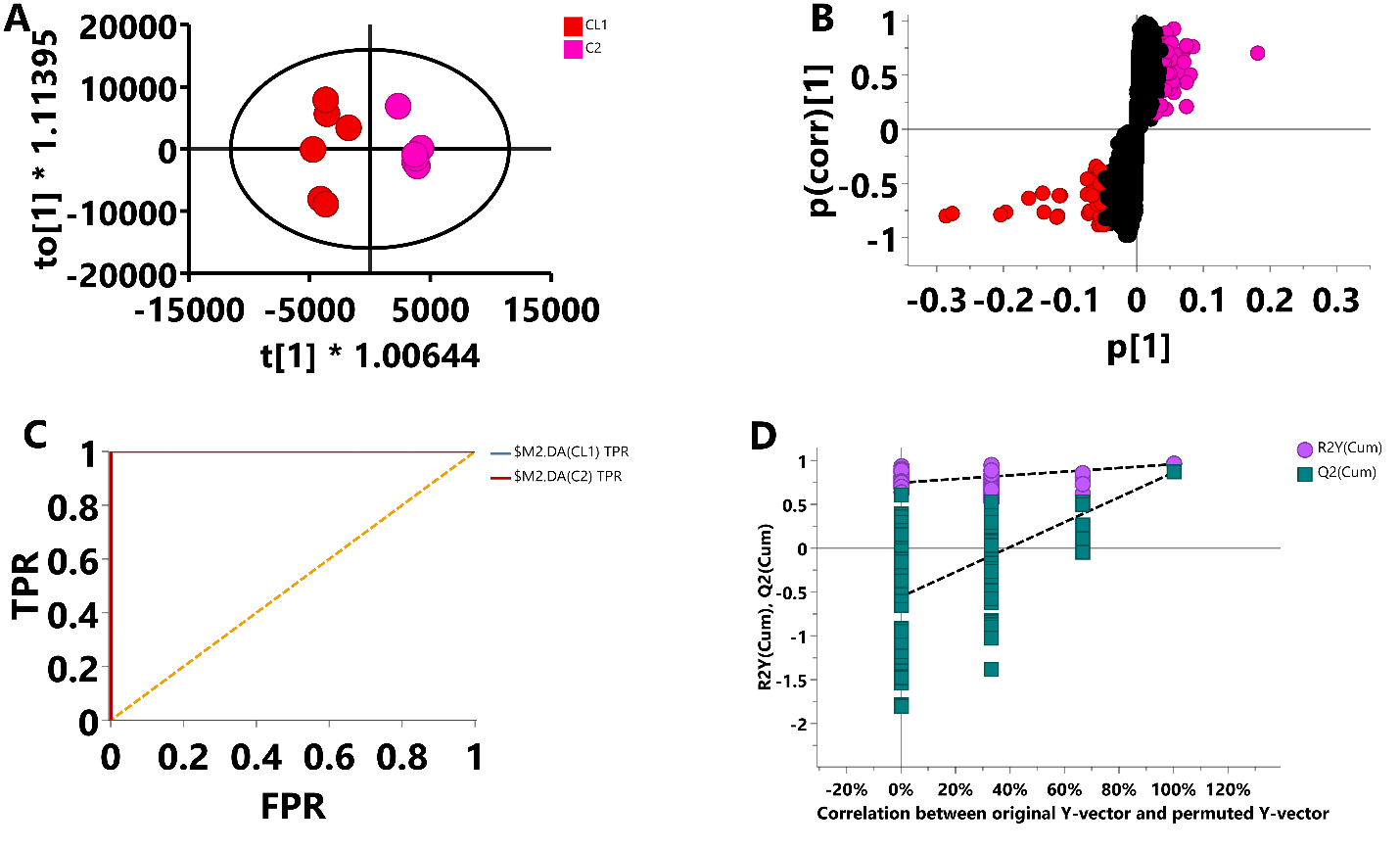


**Figure S7. Supervised orthogonal partial least squares-discriminant analysis (OPLS-DA) of maize extracts at the vegetative growth stage**. **(A**) OPLS-DA scores plot showing a clear separation between the control and consortium 2 treated samples. (**B**) OPLS-DA S-plot highlighting the key metabolic features discriminating between control and consortium treated sample (red- and pink-colored nodes). The model parameters were R^2^ X=39.4%, R^2^ Y= 95.7%, and Q^2^= 86.4%. (**C**) Receiver operator characteristic (ROC) plot summarizing the performance of the binary classified. (**D**) Permutation test plot (n = 100) validating the predictive capability of the OPLS-DA model. **Abbreviations:** CL1= control group and C2= maize treated with consortium 2.


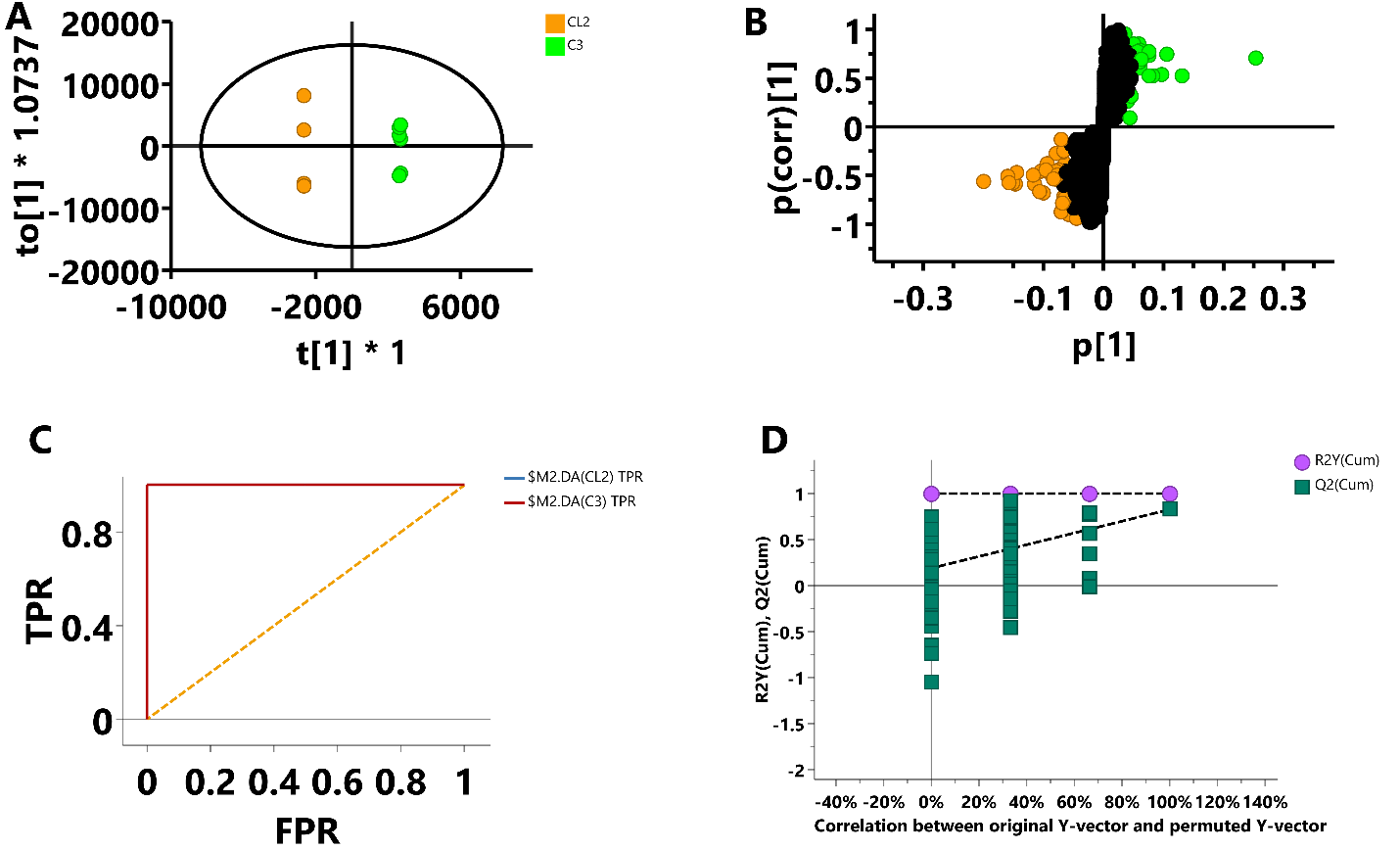


**Figure S8. Supervised orthogonal partial least squares-discriminant analysis (OPLS-DA) of maize extracts at the vegetative growth stage.** (**A**) OPLS-DA scores plot showing a clear separation between the control and consortium 3 treated samples. (**B**) OPLS-DA S-plot highlighting the key metabolic features discriminating between control and consortium treated sample (orange- and green-coloured nodes). The model parameters were R^2^ X=49.7%, R^2^Y= 1 and Q^2^= 82.7%. (**C**) Receiver operator characteristic (ROC) plot summarizing the performance of the binary classified. (**D**) Permutation test plot (n = 100) validating the predictive capability of the OPLS-DA model. **Abbreviations:** CL2= control group and C3= maize treated with consortium 3.

**Table S2. Putatively annotated discriminatory metabolites.** Putatively identified discriminating metabolites between consortia-treated groups and their respective controls. The unique discriminatory metabolites identified between treated and control groups are color-coded as follows: blue for consortium 1 treatment, pink for consortium 2 treatment, and green for consortium 3 treatment. Common discriminatory metabolites shared among all consortia and their respective controls are written in orange. Common discriminatory metabolites shared between consortia 1 and 3 treatments and their controls are written in purple. Lastly, the common discriminatory metabolite shared between consortia 2 and 3 treatments and their controls are written in red.

| **Putative annotation** | **Abbreviation** | **Molecular formula** | **Rt** | ***m/z*** | **Adduct** | **Metabolite class** |
| --- | --- | --- | --- | --- | --- | --- |
| **Sinapoyl malate** | **SinMa** | \| **C_15_H_16_O_9_** \| \| --- \| | **8.13** | **339.07** | **[M-H]-** | **HCA** |
| **9,12,13-TriHOME 2** | **9,12,13-TriHOME 2** | **C_18_H_34_O_5_** | **8.92** | **329.23** | **[M-H]-** | **Lipids** |
| **1-o-sinapoylglucose** | **1-SinAGlu** | **C_17_H_22_O_10_** | **6.22** | **385.11** | **[M-H]-** | **HCA** |
| **13-HOTrE** | **13-HOTrE** | \| **C_18_H_30_O_3_** \| \| --- \| | **10.76** | **293.21** | **[M-H]-** | **Lipids** |
| **9,12,13, TriHODE** | **9,12,13, TriHODE** | \| **C_18_H_32_O_5_** \| \| --- \| | **8.65** | **327.21** | **[M-H]-** | **Lipids** |
| **Tricin 7-diglucuronoside** | **Tri-7-diGlu** | **C_29_H_30_O_19_** | **7.01** | **681.13** | **[M-H]-** | **Flavonoid** |
| **Colnelenic acid** | **ColA** | \| **C_18_H_28_O_3_** \| \| --- \| | **11.57** | **291.19** | **[M-H]-** | **Lipids** |
| **Coumaroyl quinic acid** | **CouqA** | **C_16_H_18_O_8_** | **5.15** | **337.09** | **[M-H]-** | **HCA** |
| **Maysin** | **May** | \| **C_27_H_28_O_14_** \| \| --- \| | **7.36** | **575.13** | **[M-H]-** | **Flavonoid** |
| **2''-O-⍺-L-rhamnosyl-6-C-fucosyl-luteolin** | **2''-rhamn-fuc-lut** | **C_27_H_30_O_14_** | **11.22** | **577.26** | **[M-H]-** | **Flavonoid** |
| **2-O-p-Coumaroylhydroxycitric**  **acid** | **2-CouhydA** | \| **C_15_H_14_O_10_** \| \| --- \| | **5.65** | **353.05** | **[M-H]-** | **HCA** |
| **9-Hydroxy-12,13-epoxy-10-**  **octadecenoic acid** | **9-Hyd-epo-10** | **C_18_H_32_O_4_** | **10.96** | **311.16** | **[M-H]-** | **Lipids** |
| **Aconitic acid** | **AcoA** | \| **C_6_H_6_O_6_** \| \| --- \| | **0.974** | **173.00** | **[M-H]-** | **TCA** |
| **2-Feruloylhydroxycitric acid** | **2-FerHydA** | **C_16_H_16_O_11_** | **6.06** | **383.06** | **[M-H]-** | **HCA** |
| **Heneicosanoic acid** | **HenA** | **C_21_H_42_O_2_** | **11.49** | **325.18** | **[M-H]-** | **Lipids** |
| **3-Caffeoylquinic acid** | **3-CafqA** | **C_16_H_18_O_9_** | **5.22** | **353.08** | **[M-H]-** | **HCA** |
| **4-Caffeoylquinic acid** | **4-CafqA** | **C_16_H_18_O_9_** | **3.05** | **353.08** | **[M-H]-** | **HCA** |
| **Phosphatidylglycerol (16:0/0:0)** | **PhosG (16:0)** | **C_42_H_77_O_10_P** | **11.54** | **483.27** | **[M-H]-** | **Lipids** |
| **Caffeoylhydroxycitric acid (Isomer 1)** | **Caf-hydA 1** | **C_15_H_14_O_11_** | **3.44** | **369.04** | **[M-H]-** | **HCA** |
| **Caffeoylhydroxycitric acid (Isomer 2)** | **Caf-hydA 2** | **C_15_H_14_O_11_** | **3.78** | **369.04** | **[M-H]-** | **HCA** |
| **Caffeoylhydroxycitric acid (Isomer 3)** | **Caf-hydA 3** | **C_15_H_14_O_11_** | **4.33** | **369.04** | **[M-H]-** | **HCA** |
| **Caffeoylhydroxycitric acid (Isomer 4)** | **Caf-hydA 4** | **C_15_H_14_O_11_** | **4.57** | **369.04** | **[M-H]-** | **HCA** |
| **Apigenin-7-apioglucoside** | **Api-7-apiGlu** | **C_26_H_28_O_14_** | **9.011** | **563.13** | **[M-H]-** | **Flavonoids** |

**Table S3. p-values of the identified discriminatory metabolites shown in Figure 4.4.** Significance of the unique identified discriminatory metabolites**.**

| Unique metabolites CL2 vs C1 p-values | |
| --- | --- |
| 1-o-sinapoylglucose | 0.0905003 |
| Sinapoyl malate | 0.524669 |
| 9,12,13-TriHOME 2 | 0.112751 |
| CL1 v2 C2 p-values | |
| 13-HOTrE | 0.537805 |
| 9,12,13, TriHODE | 0.243061 |
| Tricin 7-diglucuronoside | 0.410372 |
| CL2 vs C3 p-values | |
| Coumaroyl quinic acid | 0.154322 |
| Maysin | 0.343356 |


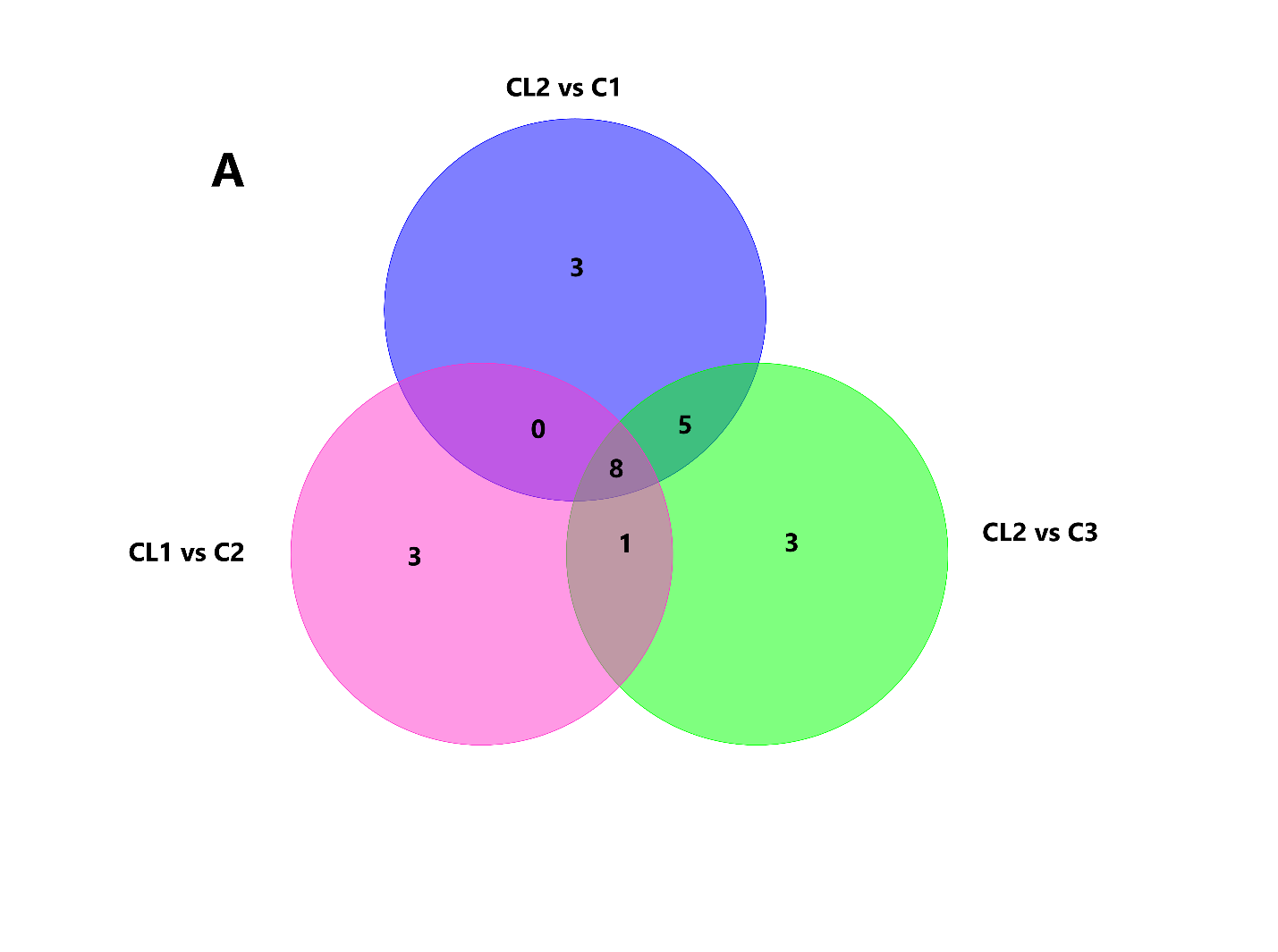


**Figure S9. Common putatively annotated extracted from orthogonal partial least squares-discriminant analysis (OPLS-DA) S-plot and their relative quantification at the vegetative stage**. Venn diagram showing common identified discriminatory metabolites (**Table S4.3**) in consortia treatments and their controls. Refer to **Table S4.3** for names of the metabolites.

**Table S4. Significantly impacted pathways.** Significantly altered metabolic pathways in consortia-treated maize plants at the different growth stages, generated from Metabolomics Pathway Analysis (MetPA).

|  | | Consortium 1 | | Consortium 2 | | | Consortium 3 | |
| --- | --- | --- | --- | --- | --- | --- | --- | --- |
| No | Pathway | Impact | -log(p) | Impact | -log(p) | Impact | | -log(p) |
| 1. | Alanine, aspartate and glutamate metabolism | 0.0036 | 0.44794 | 0.0036 | 0.097866 | 0.0036 | | 0.47635 |
| 2. | Arginine biosynthesis | 0.0 | 0.44794 | 0.0 | 0.097866 | 0.0 | | 0.47635 |
| 3. | Phenylalanine, tyrosine and tryptophan biosynthesis | 0.08008 | 0.48701 | 0.08008 | 0.8932 | 0.08008 | | 0.67698 |
| 4. | Tyrosine metabolism | 0.07027 | 0.44794 | 0.07027 | 0.097866 | 0.07027 | | 0.47635 |
| 5. | Citrate cycle (TCA cycle) | 0.06258 | 0.299 | 0.06258 | 0.34464 | 0.06258 | | 0.58821 |
| 6. | Pyruvate metabolism | 0.14351 | 0.299 | 0.14351 | 0.34464 | 0.14351 | | 0.58821 |
| 7. | Glyoxylate and dicarboxylate metabolism | 0.05758 | 0.19015 | 0.05758 | 0.63373 | 0.05758 | | 0.86225 |
| 8. | Flavone and flavonol biosynthesis | 0.0 | 0.12958 | 0.0 | 0.32214 | 0.0 | | 0.29417 |
| 9. | Flavonoid biosynthesis | 0.0322 | 0.0076948 | 0.0322 | 3.0838 | 0.0322 | | 0.93633 |
| 10 | Phenylpropanoid biosynthesis | 0.16924 | 0.024039 | 0.16924 | 3.0337 | 0.16924 | | 0.91415 |


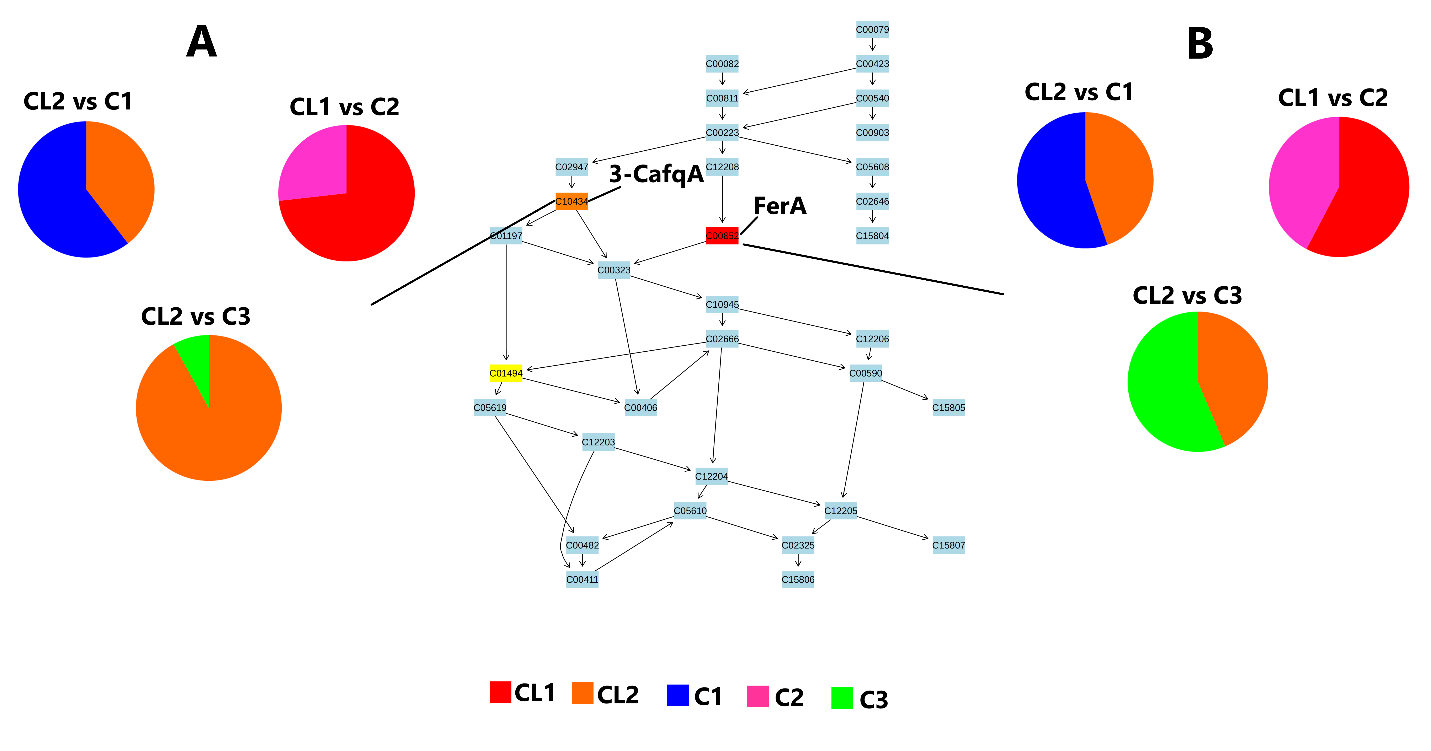


**Figure S10. Metabolic pathway analysis**. (A)The topological pathway of flavonoid biosynthesis metabolism and quantification of matched metabolite (**A**) 3-CafqA and (**B**) FerA within the pathway. Refer to **Table S4.1** for full names of metabolites. **Abbreviations:** CL1/CL2 = control, C1= maize treated with consortium 1, C2= maize treated with consortium 2, and C3=maize treated with consortium 3.
